# Establishment of in vivo proximity labeling with biotin using TurboID in the filamentous fungus *Sordaria* *macrospora*

**DOI:** 10.1038/s41598-022-22545-x

**Published:** 2022-10-22

**Authors:** Lucas S. Hollstein, Kerstin Schmitt, Oliver Valerius, Gertrud Stahlhut, Stefanie Pöggeler

**Affiliations:** 1 Department of Genetics of Eukaryotic Microorganisms, Institute of Microbiology and Genetics, Georg-August-University of Göttingen, Grisebachstr. 8, 37077 Göttingen, Germany; 2 Department of Molecular Microbiology and Genetics, Institute of Microbiology and Genetics, Georg-August-University of Göttingen, Grisebachstr. 8, 37077 Göttingen, Germany

**Keywords:** Mass spectrometry, Fungal biology

## Abstract

Proximity-dependent *bio*tin *id*entification (BioID) has emerged as a powerful methodology to identify proteins co-localizing with a given bait protein in vivo. The approach has been established in animal cells, plants and yeast but not yet in filamentous fungi. BioID relies on promiscuous biotin ligases fused to bait proteins to covalently label neighboring proteins with biotin. Biotinylated proteins are specifically enriched through biotin affinity capture from denatured cell lysates and subsequently identified and quantified with *l*iquid *c*hromatography-*m*ass *s*pectrometry (LC–MS). In contrast to many other affinity capture approaches for studying protein–protein interactions, BioID does not rely on physical protein–protein binding within native cell lysates. This feature allows the identification of protein proximities of weak or transient and dynamic nature. Here, we demonstrate the application of BioID for the fungal model organism *Sordaria* *macrospora* (Sm) using the example of the *S*TRIPAK *c*omplex *i*nteractor *1* (SCI1) of the well-characterized *str*iatin-*i*nteracting *p*hosphatase *a*nd *k*inase (SmSTRIPAK) complex as proof of concept. For the establishment of BioID in *S.* *macrospora*, a codon-optimized TurboID biotin ligase was fused to SCI1. Biotin capture of the known SmSTRIPAK components PRO11, SmMOB3, PRO22 and SmPP2Ac1 demonstrates the successful BioID application in *S.* *macrospora.* BioID proximity labeling approaches will provide a powerful proteomics tool for fungal biologists.

## Introduction

The *bio*tin *id*entification (BioID) method is an in vivo proximity-dependent labeling technique for the identification of proteins co-localizing with a given bait protein in the cell. In contrast to proximity labeling approaches relying on peroxidases, BioID does not require supplementation with H_2_O_2_ or other toxic or harmful reagents. In classical BioID experiments, a mutated variant of the *Escherichia* *coli* biotin ligase BirA (R118G, indicated as BirA*) is fused to a bait protein and locally activates biotin to biotinylate proximal proteins. BirA* catalyzes the conversion of biotin and ATP into a reactive biotinyl-5’-AMP conjugate. Due to the R118G mutation of BirA*, this reactive intermediate is released prematurely and scatters into the cytosol where it rapidly reacts with proximal primary amines of e.g. lysine residues^1,2^*.* The covalent bound biotin withstands cell lysis under denaturing conditions with SDS and lasts the enrichment procedure of labeled proteins^3^. However, the classical BirA* exhibits rather low catalytic activity and requires long time periods of labeling with culture-supplemented biotin (> 18 h)^1^. To shorten the labeling time periods, an improved biotin ligase called TurboID was developed by error-prone PCR mutagenesis and directed evolution in yeast. In total, 15 mutations were introduced into a BirA variant (R118S) to create the TurboID ligase^4^. This TurboID biotin ligase exhibits faster labeling kinetics and enables labeling durations as short as 10 min. Moreover, the TurboID ligase exhibits substantial catalytic activity at temperatures below 37 °C and according to the increased production of activated biotin, labels proteins within a somewhat extended radius compared to BirA*^5^. The BioID method has been well established in animals, plants and yeast but yet not in filamentous fungi^6–9^.

For decades, the filamentous ascomycete *Sordaria* *macrospora* (Sm) has been used as a model organism for fruiting body development of filamentous fungi^10^. *S.* *macrospora* is a haploid, homothallic (self-fertile) fungus that can sexually reproduce without another mating partner and produces exclusively sexual ascospores in fruiting bodies called perithecia. *S.* *macrospora* has a fast and simple life cycle on cheap medium. After germination of an ascospore, the life cycle is completed within 7 days under laboratory conditions. Over 100 different developmental mutant strains of *S.* *macrospora* were generated by chemical mutagenesis. Several of these mutant strains are arrested in the transition from pre-fruiting body (*pro*toperithecium) to perithecium, hence they were classified as “pro” mutants^11^. Complementation experiments of the pro11 mutant with an indexed cosmid library revealed the corresponding *pro11* gene, encoding a protein homologous to mammalian striatins^12^. Since this initial discovery of a striatin homolog in non-animal systems, more homologs have been identified in other fungal model organisms such as *Aspergillus* *nidulans* or *Saccharomyces* *cerevisiae*^13^. Striatins act as the central scaffold of the *str*iatin-*i*nteracting *p*hosphatase *a*nd *k*inase (STRIPAK) complex^14^. The STRIPAK complex is highly conserved and has been identified in various lower and higher eukaryotes^15^. It is a noncanonical multimodular protein phosphatase 2A (PP2A) complex that modulates the activity of several other conserved cellular developmental pathways, including the human Hippo pathway^16^. The human STRIPAK core complex consists of a homotetramer of the B’’’ regulatory subunit of PP2A (striatins), one copy of the scaffold subunit of PP2A (PP2AA), the catalytic subunit of PP2A (PP2AC), *s*triatin-*i*nteracting *p*rotein *1*/*2* (STRIP1/2) and *m*onopolar spindle *o*ne *b*inder *3* (MOB3). In addition, further proteins bind to the STRIPAK core to form a functional complex: *c*erebral *c*avernous *m*alformation *3* (CCM3), *g*erminal *c*enter *k*inases *III* (GCKIII) such as *m*ammalian *ST*E20-like proteins kinase *3*/*4* (MST3/4) and *s*erine/*t*hreonine-protein *k*inase *25* (STK25), *s*arco*l*emmal *m*embrane-*a*ssociated *p*rotein (SLMAP), and *s*uppressor of *IK*BK*E 1* (SIKE1)^16^. Homologs of these major mammalian STRIPAK subunits have been identified in *S.* *macrospora* (Table 1). Due to the simplicity of the lower eukaryote compared to mammalian cell cultures, the fungus provides an excellent platform for further research of the STRIPAK complex and its components. The deletion of SmSTRIPAK components in *S.* *macrospora* leads to impaired vegetative growth, defective cell fusion, and sterility due to the failure to develop mature perithecia during sexual development^13^.

**Table 1:** List of STRIPAK subunit homologs in *S.* *macrospora* and their developmental role.

STRIPAK subunit	*S.* *macrospora* homolog	Developmental process				
		Essential	Cell fusion	Fruiting body formation	Septation	Vegetative growth
PP2AA	*Smpp2aa*	×				
PP2AC	*Smpp2Ac1*		×	×		×
Striatin	*pro11*		×	×	×	
STRIP1/2	*pro22*		×	×	×	
SLAMP	*pro45*		×	×		×
SIKE1	*sci1*		×	×		×
MOB3	*Smmob3*		×	×		×
GCKIII kinases	*Smkin3/24*		×	×	×	×

Deletion of essential genes is lethal. Modified after^13^.

For the establishment of BioID in *S.* *macrospora*, a codon-optimized TurboID biotin ligase was fused to the SmSTRIPAK-component *S*TRIPAK *c*omplex *i*nteractor *1* (SCI1). Previous analysis of SCI1 reported interaction with PRO11 and PRO45^17^. Hence, this proof of concept study should identify known, as well as possibly new SmSTRIPAK components within the proteinaceous microenvironment of the SCI1-TurboID fusion protein. In the BioID experiments performed here, the known SmSTRIPAK components PRO11, SmMOB3, PRO22, and SmPP2Ac1 were found among the top hits, demonstrating that BioID was established successfully in *S.* *macrospora*.

## Results

### Validation of TurboID constructs

**Figure 1:**
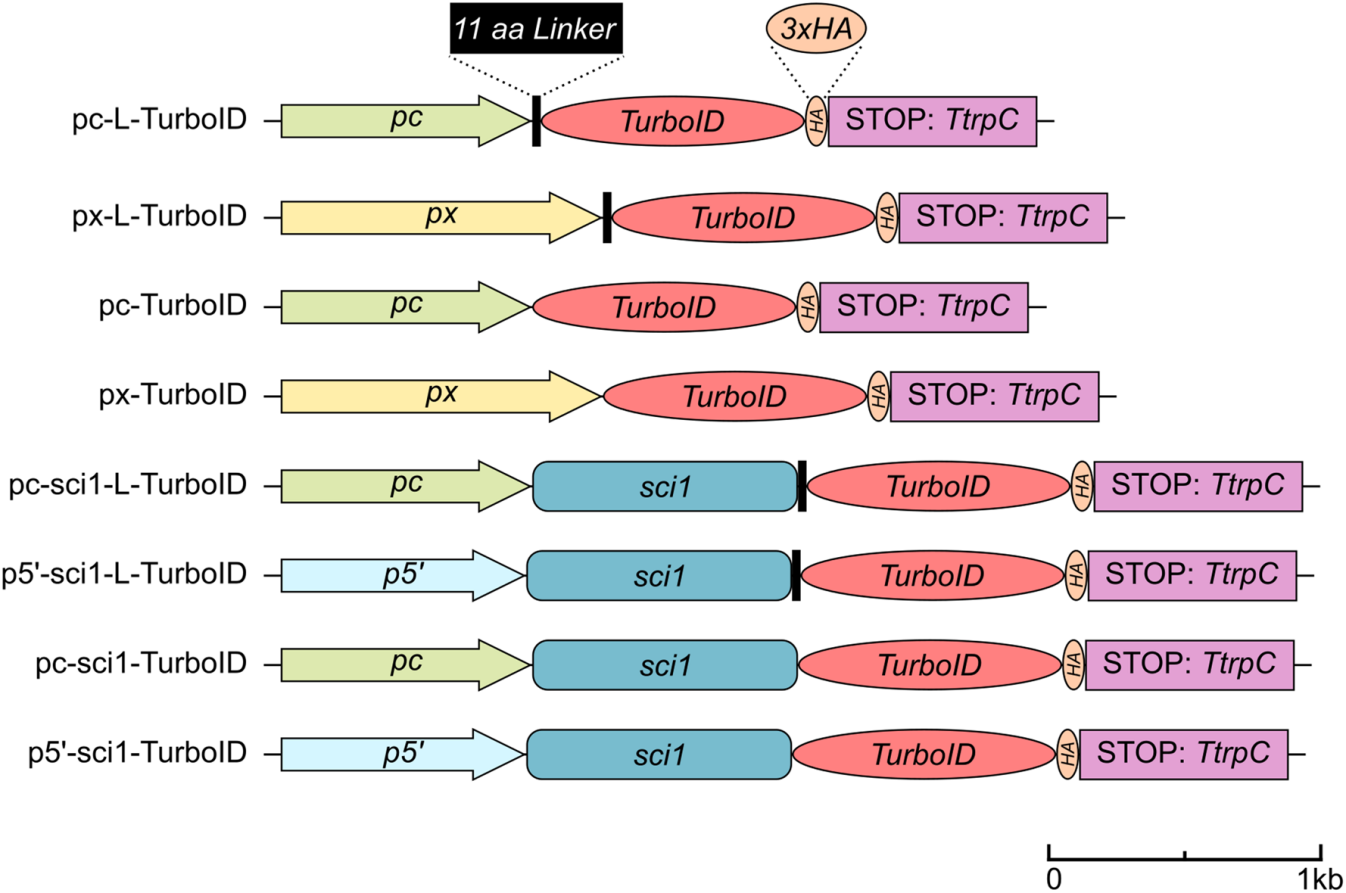
Schematic illustration of constructs used in BioID experiments. For BioID experiments, multiple constructs for the SCI1-TurboID fusion protein and the unfused TurboID control have been generated. The TurboID sequence was codon-optimized according to the codon usage table of *S.* *macrospora*^18^. Expression is controlled by different promoters: *p5’*, native *sci1* promoter; *pc*, promoter of the *clock-controlled* *gene* *1* (*ccg1*) from *N.* *crassa* for overexpression*;* *px*, xylose inducible promoter (*Smxyl*) of the *beta-xylanase* gene (*SMAC_08023*) from *S.* *macrospora*^19^*;* L, 11 amino acid linker (MGGGGSGGGGS) attached to N-terminus of TurboID; *TtrpC*, terminator of the *anthranilate* *synthase* gene from *A.* *nidulans*; a scalebar is indicated.

For the establishment of BioID in *S.* *macrospora*, multiple plasmids were generated and transformed into wild-type or the Δsci1 SmSTRIPAK mutant (Supplementary Note 1, Supplementary Tables S1–S3). These transformed strains express either the SCI1-TurboID fusion protein or an unfused TurboID ligase, the latter one serving as negative control (Fig. 1). The TurboID ligase was codon-optimized according to the codon usage table of *S.* *macrospora*^18^ (Supplementary Fig. S1). In addition, a 3xHA-tag was C-terminally fused to TurboID for convenient detection in immunoblots. Furthermore, we constructed plasmids with and without an 11 amino acid MGGGGSGGGGS linker at the N-terminus of TurboID. The expression of free TurboID is either controlled by the *ccg1* constitutive promoter (pc) of the *clock-controlled* *gene* *1* from *Neurospora* *crassa* or the xylose inducible *Smxyl* promoter (px) of the *beta-xylanase* gene (SMAC_08023) from *S.* *macrospora*^19^. Either the native *sci1* promoter (p5’) or the constitutive *ccg1* promoter controls expression of the SCI1-TurboID fusion protein in the Δsci1 strain.

**Figure 2:**
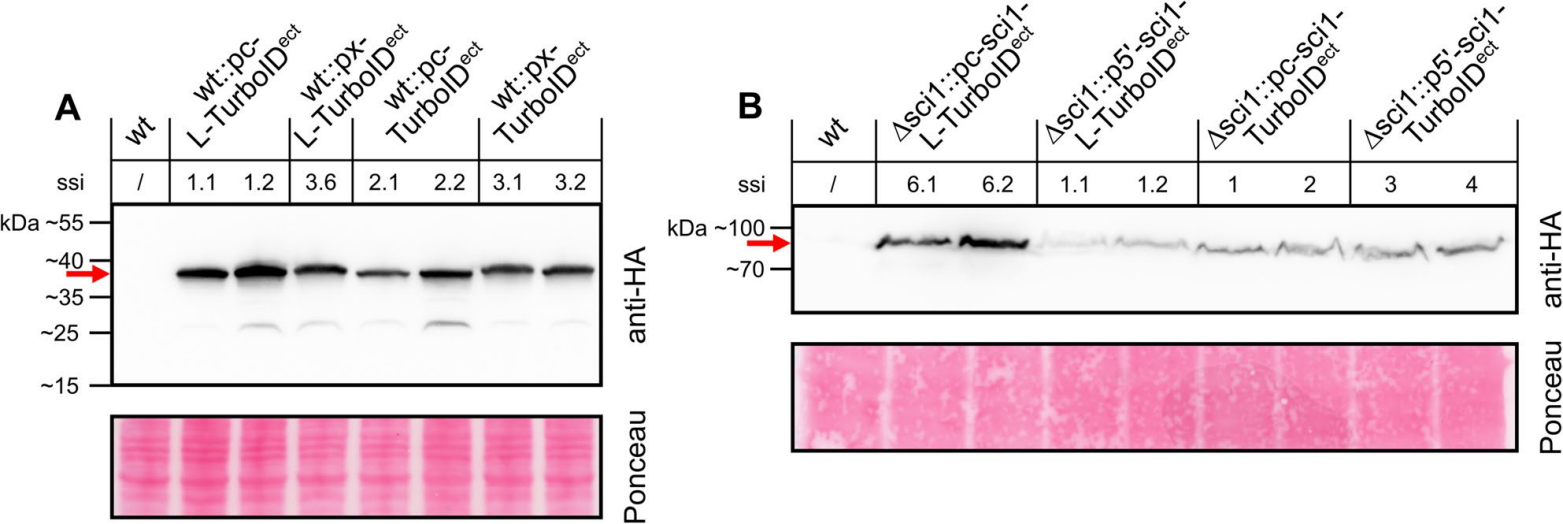
Western blot detection of TurboID constructs using an anti-HA antibody. Expression of the (**A**) free TurboID and (**B**) SCI1-TurboID fusion proteins in *S.* *macrospora* wt and Δsci1 was determined by Western blot hybridization using a monoclonal anti-HA antibody, which detects the C-terminal 3xHA tag of TurboID. Red arrows indicate (**A**) free TurboID (38.5 kDa) and (**B**) SCI-TurboID fusion proteins (71.6 kDa). Expression of the constructs is controlled by either the *ccg1* overexpression promoter from *N.* *crassa* (pc), the native *sci1* promoter (p5’), or the xylose inducible *Smxyl* promoter (px)^19^. Untransformed wt was used as negative control. *S.* *macrospora* strains were grown in liquid BMM for 3 days at 27 °C. 18 µL of crude protein extract were loaded, and Ponceau red staining was used as loading control. Blots and Ponceau red staining in (**A,B**) show the identical membrane, exposure was identical for all parts of the gel. Figure shows cropped blots. Full length blots and Ponceau red staining are depicted in Supplementary Fig. S2. ect, *ect*opically integrated; ssi, *s*ingle-*s*pore *i*solate.

Based on the amino acid sequence, the expected molecular masses were calculated for free TurboID (38.5 kDa), the SCI1-TurboID fusion protein (71.6 kDa), and the linker (0.8 kDa). To verify expression and compare the protein levels among the free TurboID and SCI1-TurboID strains, all available strains were grown in liquid *B*io*m*alt *m*aize (BMM) medium for 3 days at 27 °C. After protein extraction, the crude protein extract was separated by SDS–*p*oly*a*crylamide *g*el *e*lectrophoresis (SDS-PAGE) and immunoblotted using a monoclonal anti-HA antibody for signal detection (Fig. 2, Supplementary Fig. S2).

The free TurboID ligase and the SCI1-TurboID fusion proteins were detected at their expected molecular masses in the Western blot experiment (Fig. 2). In addition, the expression of TurboID was verified with an anti-BirA antibody (Supplementary Fig. S3).

For the SCI1-BioID experiments we decided to use the native *sci1* promoter for authentic expression regulation to remain at the most physiological conditions for the SCI1-BioID experiments. The functionality of the SCI1-TurboID fusion protein was confirmed by complementation of a *S.* *macrospora* Δsci1 mutant. Deletion of *sci1* causes sterility due to the inability to develop fruiting bodies^17^. Hence, the transformation of Δsci1 with SCI1-TurboID constructs should rescue this deficiency and restore fertility. For the complementation analysis Δsci1, Δsci1::p5’-sci1-TurboID^ect^ and Δsci1::p5’-sci1-L-TurboID^ect^ were grown on solid medium. Both deletion strains expressing the TurboID-tagged *sci1* gene were complemented and developed mature fruiting bodies, which indicates that the SCI1-TurboID and SCI1-L-TurboID fusion proteins can fulfill the endogenous function of SCI1. Sexual structures such as female ascogonia and protoperithecia developed in the complemented deletion mutant in the same time as in the wild-type (Supplementary Fig. S4). Furthermore, the linker did not affect the complementation (Supplementary Fig. S5).

**Figure 3:**
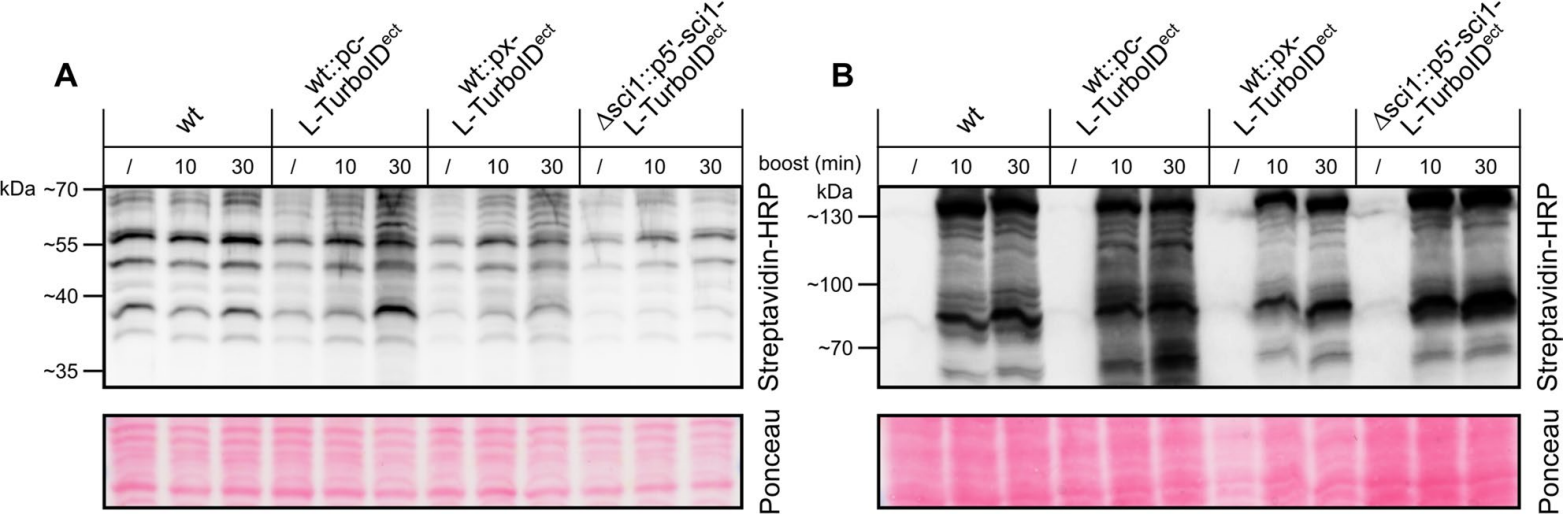
Western blot analysis of TurboID activity in recombinant *S.* *macrospora* strains. The activity of free TurboID and SCI1-TurboID fusion proteins in *S.* *macrospora* was determined by Western blot-like hybridization using Streptavidin-HRP. Streptavidin binds biotinylated proteins, and HRP is used for signal detection via chemiluminescence. Expression of the constructs is controlled by either the *ccg1* overexpression promoter from *N.* *crassa* (pc), the native *sci1* promoter (p5’), or the xylose inducible *Smxyl* promoter (px)^19^. Untransformed wt shows endogenous biotinylation of *S.* *macrospora*. 2.25 µL crude protein extract were loaded onto the gel. Ponceau red staining was used as loading control. A) Strains were grown in liquid BMM medium for 3 days at 27 °C. Before harvest, BMM supplemented with biotin was added (final concentration = 410 nM) for 10 or 30 min. B) Strains were grown in liquid SWG medium (1 g/L arginine, without biotin) for 5 days at 27 °C. Shortly before harvest, SWG supplemented with biotin was added (final concentration = 410 nM) for 10 or 30 min. Blots and Ponceau red staining in (**A,B**) show the identical membrane, exposure was identical for all parts of the gel. Figure shows cropped blots. Full length blots and Ponceau red staining are depicted in Supplementary Fig. S6. ect, *ect*opically integrated.

Finally, the ligase activity of TurboID was analyzed. Selected strains were grown in liquid BMM complex medium and synthetic *Sordaria* *W*ester*g*aard’s medium (SWG) without biotin for 3 and 5 days at 27 °C, respectively. Shortly before harvest, exogenous biotin was added to the liquid cultures to increase the potential of protein labeling proximal to the bait. This biotin boost was provided for 10 or 30 min with biotin solved in either BMM or SWG medium to a final concentration of 410 nM. A non boosted sample served as control and represented the level of biotinylation occurring throughout the growth without biotin boost. Strains grown in BMM were harvested after 3 days, whereas cultivation in SWG (without biotin) had to be prolonged to 5 days due to extremely low yields of mycelium. A Streptavidin-HRP conjugate was used for the detection of biotinylated proteins (Fig. 3, Supplementary Fig. S6).

*S.* *macrospora* strains grown in complex BMM medium exhibit biotinylated proteins even without the supplementation of exogenous biotin during boosting. Supplementation of the untransformed wild-type strain with biotin led to a slight increase in natural protein biotinylation. Overexpression of the free TurboID by the *ccg1* promoter resulted in background biotinylation after 10 min, which increased even further after 30 min of boosting. As this substantial increase in biotinylation was not detectable in the wild-type strain, this increased biotinylation is caused by the activity of TurboID. Expression of TurboID under control of the xylose inducible *Smxyl* promoter yields biotinylation levels similar to those in wild-type cells. When TurboID was fused to SCI1 and expression controlled by the *sci1* promoter, only a slight increase in biotinylation was observed, similar to the biotinylation in wild-type cells. Hence, it is unclear whether the biotinylation in Δsci1::p5’-sci1-L-TurboID^ect^ is the result of endogenous ligases or the SCI1-TurboID fusion protein (Fig. 3A).

When grown in synthetic SWG medium without biotin, biotinylated proteins were mostly absent (Fig. 3B). Strains grown in SWG medium and boosted with biotin show similar biotinylation patterns as in BMM medium. Overexpression of free TurboID led to strong biotinylation upon boosting. In contrast, expression of free TurboID from the *Smxyl* promoter and expression of SCI1-TurboID under control of the native *sci1* promoter result in wild-type-like biotinylation levels after exogenous supplementation of biotin. In both media, biotinylation levels were higher when the boosting duration was prolonged from 10 to 30 min. Therefore, boosting in the following experiments was performed for 30 min.

We observed that growth of *S.* *macrospora* in SWG medium without biotin is not feasible for further experiments, as mycelium yields were extremely low and sexual development was inhibited. These results indicated that *S.* *macrospora* might be biotin auxotroph. *B*asic *L*ocal *A*lignment *S*earch *T*ool (BLAST) searches with fungal genes of the biotin synthesis pathway revealed no homologs of de novo biotin biosynthesis genes in the genome of *S.* *macrospora.* Only a homolog of high-affinity plasma membrane H^+^-biotin (vitamin H) symporter SmVHT1 is encoded by *S.* *macrospora.* This result confirmed that *S.* *macrospora* relies on exogenous biotin supplementation for growth and development (Supplementary Fig. S7).

For BioID experiments in *S.* *macrospora*, the following criteria were set for the desired medium: 1) full complementation of the life cycle, as indicated by discharged ascospores after 7–9 days; 2) low concentration of biotin to avoid potential side effects of unspecific biotinylation by the TurboID ligase during vegetative growth. To find a suitable biotin concentration, *S.* *macrospora* was grown on SWG with 410, 200, 100, or 50 nM biotin (Supplementary Fig. S8). Based on these results, strains for BioID experiments were grown in liquid SWG medium supplemented with 50 nM biotin for 4 days at 27 °C. Shortly before harvest of the mycelium, the cultures were boosted with SWG supplemented with 10 µM biotin for 30 min at RT.

### TurboID proximity proteomics with the fusion protein SCI1-L-TurboID

**Figure 4:**
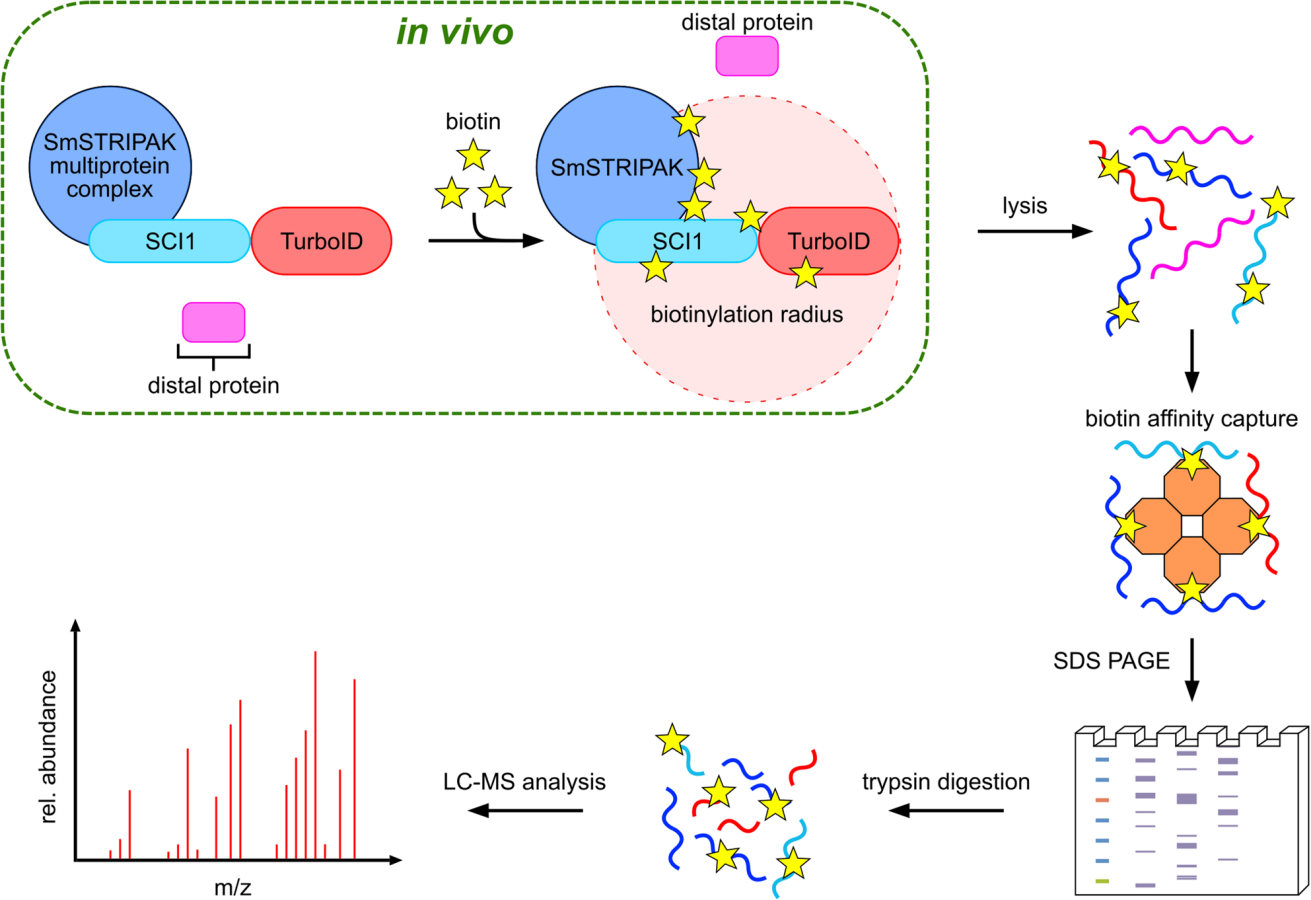
Workflow of BioID experiment with SCI1 as bait. For the establishment of BioID and identification of putative protein interaction partners of the SmSTRIPAK complex, the TurboID biotin ligase was fused to the bait protein SCI1 (C-terminally). Exogenous addition of biotin (yellow stars) leads to promiscuous biotinylation of proteins in the proximity of the SCI1-TurboID fusion protein. Distant proteins are not biotinylated. After 30 min of labeling, the cells are lysed, denatured, and biotinylated proteins are purified through Strep-Tactin Sepharose beads. The proteins are separated by SDS-PAGE. Peptides for LC–MS analysis are obtained by tryptic in-gel digestion.

The workflow of a BioID experiment with SCI1 as bait fused to TurboID is depicted in Fig. 4. To reliably identify SCI1 proximal proteins and putative physical interactors of the SmSTRIPAK protein SCI1, the BioID experiment was performed with four biological replicates of Δsci1::p5'-sci1-L-TurboID^ect^ (*s*ingle-*s*pore *i*solates (ssi) 1.3, 1.6, 1.9, 1.10) and wt::pc-L-TurboID^ect^ (ssi 1.7, 1.8, 1.9, 1.11). As the expression of free TurboID by the *ccg1* overexpression promoter results in high biotinylation levels, the crude protein extract of the control strain was diluted 1:6 with extract of the wild-type strain (non boosted) to match the biotinylation level of the Δsci1::p5'-sci1-L-TurboID^ect^ strain. Biotinylated proteins were enriched using Strep-Tactin Sepharose beads and eluted proteins were afterwards separated with SDS-PAGE. Proteins were digested with trypsin in-gel prior to *l*iquid *c*hromatography-*m*ass *s*pectrometry (LC–MS) analysis.

**Figure 5:**
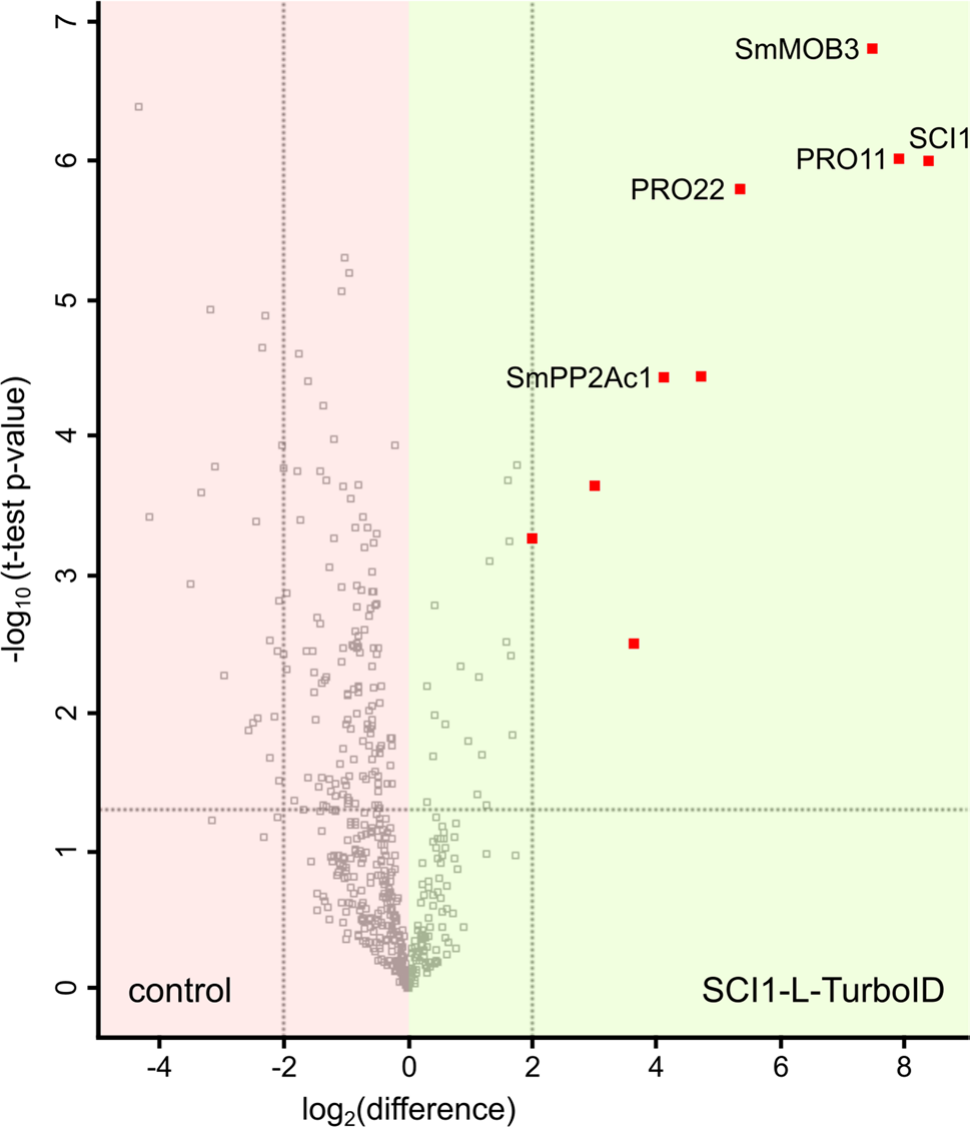
Volcano plot of proteins identified in the SCI1-BioID experiment. The volcano plot depicts the differences of log_2_ transformed LFQ intensities between Δsci1::p5'-sci1-L-TurboID^ect^ and the control (wt::pc-L-TurboID^ect^ diluted 1:6 with wild-type crude protein extract) on the x-axis. The −log_10_(p-value) is plotted on the y-axis. The gray dotted threshold lines indicate a p-value of 0.05 (horizontal) and a log_2_(difference) of −2 or + 2 (vertical). Missing values were imputed in four repetitions. Proteins that were significantly enriched (log_2_(difference) ≥ 2) in the SCI1-L-TurboID strain in all four imputations are marked with a red square.

The reproducibility of the four biological replicates from both strains was high, as indicated by mean Pearson correlations of 0.9556 among Δsci1::p5'-sci1-L-TurboID^ect^ replicates, and 0.9676 among the control replicates. A total of 1185 proteins were identified. The *L*abel *F*ree *Q*uantification (LFQ) intensities and MS/MS counts of proteins significantly enriched against the negative control (enrichment factor ≥ 4) are listed in Table 2. The SmSTRIPAK components SCI1, PRO11, SmMOB3, PRO22, and SmPP2Ac1 were identified among the top hits. One peptide of SmPP2Ac1 was found to be biotinylated at residue K308. The volcano plot in Fig. 5 shows SmSTRIPAK components identified relative to the other enriched proteins dependent on statistical significance and log_2_(difference) values as described in Supplementary Table S4. Uncharacterized candidate proteins were identified using the UniProtKB BLAST. The BLAST results are listed in Supplementary Table S5.

**Table 2:**
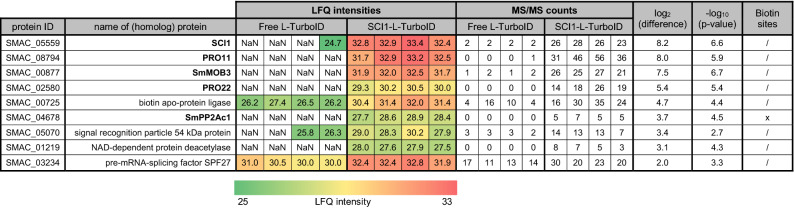
Significantly enriched proteins co-localizing with SCI1-L-TurboID in BioID.

This BioID experiment was performed with the *S.* *macrospora* strains Δsci1::p5'-sci1-L-TurboID^ect^ (SCI1-L-TurboID) and wt::pc-L-TurboID^ect^ (free L-TurboID, control). Four biological replicates each were grown in liquid SWG (50 nM biotin) for 4 days at 27 °C under continuous light conditions. Significant proteins (enrichment factor ≥ 4) were selected from a volcano plot (two-sample Student’s T-test; FDR = 0.01; s0 = 2) after filtering for 50% valid values in total. Missing values were imputed in 4 replicates. log_2_(difference) and -log_10_(p-values) are means of these 4 imputations. Proteins are sorted in descending order based on the log_2_(difference) values. LFQ intensities are color-coded: high values are shaded in red and low intensities are shaded green. Bold proteins indicate known components of the SmSTRIPAK complex. Protein names were identified through the UniProtKB BLAST (for identity % and e-values see Supplementary Table S5). [Sm], *Sordaria* *macrospora;* [Nc], *Neurospora* *crassa*; [Nt], *Neurospora* *tetrasperma*; [Mm], *Madurella* *mycetomatis.*

In addition to the known SmSTRIPAK components, 4 other proteins were significantly enriched: a putative biotin apo-protein ligase (SMAC_00725), a signal recognition particle 54 kDa protein (SMAC_05070), a putative NAD-dependent protein deacetylase (SMAC_01219), and a putative pre-mRNA-splicing factor SPF27. No phosphorylation sites (STY) were identified among these top hits. A list of all 1185 identified proteins, their quantitative values, number of peptides and sequence coverage is shown in Supplementary Dataset S1.

## Discussion

The aim of this work was to establish the BioID method for the identification of in vivo bait-proximal proteins and putative interaction partners in the filamentous ascomycete *S.* *macrospora*. As a case study, we used the SmSTRIPAK component SCI1 as bait protein fused to the TurboID ligase. We demonstrated the functionality of the SCI1-TurboID fusion protein by its capability to complement the defects of a Δsci1 mutant^17^.

Biotin supplementation proved to be an important factor controlling the outcome of a BioID experiment. Without efficient generation of activated biotin within the bait’s microenvironment, proximal proteins are not efficiently captured during biotin affinity purification. However, excessive biotinylation by the TurboID ligase may reduce the spatial narrowness due to an increased diffusion cloud of activated biotin. Moreover, potential side effects may arise from an abundant non-physiological biotinylation^20^. Anyway, *S.* *macrospora* is biotin auxotroph and therefore depends on external biotin supply to complete its life cycle. Therefore, in contrast to yeast, biotin supplementation cannot be restricted to the short boosting time period prior to the harvest of the mycelium.

The BioID candidates of the SCI1-TurboID experiment are supposed to overlap with previous SmSTRIPAK complex pulldown experiments (Table 3). Surprisingly, with the SCI1-BioID approach more components of the complex were identified in a single run than with the previous pulldowns and TAP/MS experiments^17,21,22^ (Table 3). Only pulldown experiments with PRO11 identified a similar number of SmSTRIPAK subunits^17^. As PRO11 is the main complex scaffold whereas SCI1 is considered as a less integral component of the complex, these results underline the power and efficiency of the BioID approach in the identification of proteinaceous microenvironments.

**Table 3:** Comparison of SCI1-BioID candidates with previous studies investigating SmSTRIPAK interactors.

Candidates identified in SCI1-BioID		Phosphoproteome studies		Pulldown experiments			TAP/MS experiments	
		Märker et al. (2020)^23^	Stein et al. (2020)^24^	Reschka (2018)^25^			Bloemendal et al. (2012)^21^	Nordzieke et al. (2015)^22^
Protein ID	Name of (homolog) protein	Regulation	Regulation	PRO11	SmMOB3	SCI1	PRO22	PRO45
SMAC_05559	**SCI1 [Sm]**	–	–	×	–	×	–	×
SMAC_08794	**PRO11 [Sm]**	–	–	×	×	×	×	×
SMAC_00877	**SmMOB3 [Sm]**	–	×	×	×	–	–	×
SMAC_02580	**PRO22 [Sm]**	–	–	×	–	–	×	–
SMAC_00725	Biotin apo-protein ligase [Nc]	Not identified	Not identified	–	–	–	–	–
SMAC_04678	**SmPP2Ac1 [Sm]**	Not identified	not identified	–	–	–	×	–
SMAC_05070	signal recognition particle 54 kDa protein [Sm]	Not identified	not identified	–	–	–	–	–
SMAC_01219	NAD-dependent protein deacetylase [Sm]	–	–	–	–	–	–	–
SMAC_03234	pre-mRNA-splicing factor SPF27 [Mm]	Not identified	not identified	–	–	–	–	–

Significantly enriched proteins of the SCI1-BioID experiment (enrichment factor ≥ 4) were compared to the results of phosphoproteome studies^23,24^, pulldown^25^ and TAP/MS experiments^21,22^ with SmSTRIPAK subunits in *S.* *macrospora.* For phosphoproteome analysis, the SmSTRIPAK single and double subunit deletion strains ΔSmpp2Ac1, Δpro11, Δpro22^23^ and Δpro11, Δpro11Δpro22, ΔSmpp2Ac1Δpro22^24^ were used. Peptides were labeled using the iTRAQ approach for relative quantification and phosphopeptides were enriched using TiO_2_. Phosphopeptides with a Log_2_ ratio two times higher than the standard deviation of the total dataset of the given protein were considered regulated. Proteins with regulated phosphopeptides in at least one knockout strain are marked with an “×” in the “regulation” column. Downregulations of phosphopeptides in the associated deletion strain were ignored (e.g., downregulation of SmPP2Ac1 phosphopeptides in ΔSmPP2Ac1). Pulldown experiments were carried out using HA-PRO11, HA-PRO11-eGFP, FLAG-SmMOB3, SmMOB3-eGFP and SCI1-eGFP as bait. Criteria for enrichment were: at least two peptides per protein, peptide mass confidence high, not enriched in negative control and at least seven spectral counts in two out of three biological replicates^25^. TAP/MS experiments were performed with PRO22^21^ and PRO45 as bait^22^. For TAP/MS experiments, only proteins identified in all biological replicates were marked with “×”. Protein names were identified through the UniProtKB BLAST (for identity % and e-values see Supplementary Table S5). [Sm], *Sordaria* *macrospora*; [Nc], *Neurospora* *crassa*; [Nt], *Neurospora* *tetrasperma*; [Mm], *Madurella* *mycetomatis.*

The SmSTRIPAK components SCI1, PRO11, SmMOB3, PRO22, and SmPP2Ac1 were found with high MS/MS counts in the strain expressing SCI1-L-TurboID, and they were almost absent in the free TurboID control. In previous yeast-two-hybrid (Y2H) assays, direct interaction of SCI1 with PRO11 and PRO45 has been reported. However, SCI1 did not interact with PRO22 in these Y2H experiments^17^. Thus, the identification of PRO22 with the BioID approach illustrates the identification of proteins that might not physically contact but co-localize with the bait protein within the same molecular microenvironment. Moreover, the identified SmSTRIPAK components are members of the group of most valid proteins reported in the experiment. The very high enrichment values of PRO11 and SCI1 may be caused by their stochiometric arrangement involving multiple monomers. SCI1 was shown to homodimerize, which may enable extensive trans‑biotinylation of the respective monomers^17^. Apart from the dimerization, the TurboID ligase is expected to biotinylate its fused bait protein. Similar to the human homolog, PRO11 might form a homotetramer when incorporated into the SmSTRIPAK complex^16^. As a result, a single SCI1-L-TurboID protein may biotinylate multiple PRO11 copies. In addition to the significant enrichment of PRO11, SmMOB3, PRO22 and SmPP2Ac1, the PP2A activator PTPA1 (SMAC_03446) was slightly enriched in the SCI1-L-TurboID expressing strain (log_2_(difference) = 1.6; −log_10_(p-value) = 3.69). This activator has been previously shown to interact with SmPP2Ac1 in Y2H experiments^26^. Analysis of the human and *S.* *cerevisiae* PTPA homologs revealed that the regulator is required for proper maturation and reactivation of the inactive catalytic subunit PP2Ac to avoid unspecific phosphatase activity of PP2Ac in its unbound form^27–29^. For activation of PP2Ac, PTPA physically binds and acts as an ATP‑dependent chaperone by stabilizing the conformation of apo-PP2Ac. Additionally, PTPA was shown to reduce partial unfolding and aggregation of apo-PP2Ac^30^.

Unexpectedly, no peptides of PRO45 were detected, which raises several questions. An interaction between SCI1 and PRO45 has been demonstrated in *S.* *macrospora* and their homologs in *H.* *sapiens* and *A.* *nidulans*^17,31–33^. In vitro phosphorylation assays with the human PRO45 homolog SLMAP and the SCI1 homolog SIKE1 demonstrated a crucial role of the SLMAP-SIKE1 heterodimer for STRIPAK functionality. The phosphatase activity of the STRIPAK was substantially increased in the presence of the SLMAP-SIKE1 heterodimer^16^. Previously we were able to demonstrate a direct interaction of PRO45 and SCI1. In Y2H experiments, PRO45 strongly interacted with the N-terminal half, but not the C-terminal half of SCI1^17^. Hence, it seems unlikely that the fusion of TurboID to the C-terminus of SCI1 disrupted the interaction with PRO45, especially regarding the complementation of sterility in Δsci1 by the SCI1-TurboID fusion protein. Intriguingly, pulldown experiments with PRO11 as bait could not detect PRO45^17^, whereas TAP/MS using PRO45 as bait identified PRO11, SmMOB3, and SCI1^22^. Moreover, PRO45 was not identified in TAP/MS experiments with PRO22 as bait^21^. The absence of PRO45 detections might be explained by its C-terminal transmembrane domain and it’s localization at the nuclear envelope, the ER, and mitochondria^22^. While cytosolic proteins can be extracted using simple procedures, extraction of membrane proteins is more complex and challenging. Possibly the protein preparation used in this study does not allow effective isolation of membrane proteins. Furthermore, no proteins of the nuclear envelope were identified, although many components of the SmSTRIPAK complex, including SCI1, were shown to localize to the nuclear membrane^13,17^.

The significant enrichment of the biotin apo-protein ligase (SMAC_00725) might be caused by the control used in the SCI1-BioID experiment. It is conceivable, that supplementation of exogenous biotin increases the expression of the biotin apo-protein ligase. In this case, the dilution of the control strain overexpressing unfused TurboID with crude protein extract of the wild-type (not boosted with biotin) might result in an artificial underrepresentation of biotin apo-protein ligase peptides in the control samples.

The significant enrichment of the pre-mRNA splicing factor SPF27 could be explained by a possible link of the SmSTRIPAK complex to endosomal mRNA transport. Phosphoproteome analyses of single and double SmSTRIPAK deletion strains detected SmSTRIPAK-dependent phosphorylation of eight proteins with RNA recognitions motifs. One of these proteins, GUL1, is an RNA-binding protein that shuttles on early endosomes and might participate in endosomal mRNA transport along microtubules^24^. Interestingly, the *Ustilago* *maydis* homolog of the pre-mRNA splicing factor SPF27, Num1 was shown to interact with the microtubule-associated motor protein kinesin KIN1. The *num1* deletion strain exhibits an impaired transport of early endosomes and in addition to its involvement in splicing in the nucleus it is suggested to have further functions in the cytosol in connection with KIN1^34,35^. However, none of the other significantly enriched proteins of the SCI1-BioID seem to be involved in RNA binding.

Currently, there is no explanation for the significant enrichment of the signal recognition particle 54 kDa protein and the NAD-dependent protein deacetylase. These proteins have not been reported in context with SCI1 or the SmSTRIPAK complex, yet. Further experiments are needed to determine whether these proteins co-localize with the SmSTRIPAK or if they were artificially enriched.

Overall, our results demonstrate that TurboID can be used to identify biologically relevant proximal proteins of fused bait proteins in *S.* *macrospora*. The plasmids generated and biotin-labelling methods established in this study should prove applicable in a wide variety of filamentous fungi as well, and provide a new tool for the identification of novel protein–protein interactions in filamentous fungi.

## Methods

All *S.* *macrospora* strains, primers and template plasmids used for PCR amplifications as well as all plasmids generated in this study are listed in Supplementary Tables S1, S2 and S3, respectively. A detailed description of strains, media, growth conditions, transformation of *S.* *macrospora*, generation of single-spore isolates, protein extraction as well as the generation of plasmids can be found in the Supplementary Note 1.

### Western blot and immunodetection

For the immunodetection of proteins, the proteins were transferred onto a Protan BA85 nitrocellulose membrane (Cytiva) using the Semi-dry Xpress Blotting kit (Serva) according to the instructor’s manual after SDS-PAGE. Blotted proteins were visualized using 0.2% Ponceau S in 3% TCA solution. After documentation, the membrane was fully destained through washing with TBST and the membrane was blocked using 5% milk powder in 1 × TBST. Membranes for the analysis of biotinylated protein through Streptavidin-HRP were blocked overnight at 4 °C, washed 3 × 15 min with 1× TBST, and incubated 50 min with Streptavidin-HRP [Thermo Scientific (21130), 1:30,000]. Expression analysis of free TurboID and SCI1-TurboID fusion proteins was performed using an anti-HA (Sigma-Aldrich (H9658), 1:3000) or anti-BirA antibody (antibodies-online GmbH (ABIN2691501), 1:2000). Subsequently, membranes were incubated with secondary HRP-coupled antibodies (HRP-linked anti mouse, Sigma-Aldrich (A5278), 1:10,000; HRP-linked anti rabbit, Thermo Scientific (G21234), 1:5000) for 60 min in darkness. For detection of a luminescent signal, the membranes were covered in 500 µL WesternBright ECL HRP (Advansta Corporation) substrate. Chemiluminescence was detected and documented using the digital Vilber Lourmat FUSION SL imaging system (PEQLAB Biotechnologie GmbH).

### Biotin affinity purification using Strep-Tactin Sepharose

For the BioID experiment with Δsci1::p5'-sci1-L-TurboID^ect^, four independent biological replicates were cultivated in 50 ml SWG (50 nM biotin, 40 mg/L arginine) for 4 days at 27 °C under continuous light conditions. Before harvest, the strains were boosted by adding 20 ml SWG supplemented with 30 µM biotin for 30 min. Due to evaporation throughout the 4 days of growth, roughly 40 ml of the initial SWG medium remained in the Petri dishes. Therefore, boosting with 20 ml medium results in a final biotin concentration of 10 µM. The wt::pc-L-TurboID^ect^ strain, which expresses an unfused TurboID, was used as control in this experiment. As the *ccg1* overexpression promoter controls the expression of the free TurboID ligase in the control strain, this strain exhibits stronger biotinylation levels than the strain expressing the SCI1-L-TurboID fusion protein. Thus, the crude protein extract of the control was diluted 1:6 with wild-type extract (not boosted with biotin) to match the biotinylation level of the Δsci1::p5'-sci1-L-TurboID^ect^ strain. After protein extraction, 800 µL crude protein extract were mixed with 200 µL 20% SDS to obtain a final concentration of 4% SDS. The samples were denatured by heating to 65 °C for 5 min. Biotinylated proteins were enriched using Strep-Tactin Sepharose (IBA Lifesciences GmbH) similar to Schmitt and Valerius (2019)^36^. In brief, 100 µL of slurry were equilibrated per sample by two washing steps with 375 µL 1× Buffer W. Then, 1 ml denatured protein extract was added to the beads and incubated on a rotating wheel for 30 min at RT. Afterwards, the crude protein extract containing unbound biotinylated proteins was separated from the beads by centrifugation, and the beads were washed three times with 1× Buffer W (+ 0.4% SDS). Next, biotinylated proteins were eluted by adding 75 µL 1× Buffer BXT and 10 min incubation on the shaker. The supernatant containing the biotinylated proteins was collected by centrifugation at 400×*g* for 2 min. This elution step was performed twice for a total of 150 µL eluate. Eluates for in gel digestions were entirely concentrated in the SpeedVac and resuspended in TE Buffer and 2 × Tris–Glycine/SDS sample buffer. The whole eluate was loaded onto a 12% polyacrylamide gel and was run until the bromophenol of the loading dye migrated through roughly 1/3 of the resolving gel. The gels of the BioID experiments were incubated in fixing solution for 30 min, and the lanes of interest were excised using a clean scalpel. Each lane was fractioned in approximately 2 mm big pieces, which were stored in 1.5 ml Protein LoBind tubes filled with A. dest.

### Trypsin in-gel digest and C18 STAGE tip purification

The tryptic in-gel digestion, C18 STAGE tip purification and LC–MS analysis were performed as previously described in Groth et al. (2021)^37^.

### Analysis of LC–MS raw data

LC-MS raw files were analyzed using the MaxQuant (version 1.6.10.43) and Perseus (version 1.6.0.7) software^38,39^. MS raw data files were searched against the *S.* *macrospora* specific proteomics data set Smacrospora_v03_peptides.fasta from Blank-Landeshammer et al. (2019)^40^ with max missed cleavage sites = 3. Oxidation of methionine, N-terminal acetylation of proteins, Phosphorylation (STY) and biotinylation of lysines were set as variable modifications. “Trypsin/P” was used as digestion mode. Samples were quantified by label-free quantification. The decoy sequences were generated by the revert mode, and the false discovery rate (FDR) was set to 0.01. The MaxQuant output file “proteinGroups.txt” was used for further data analysis in Perseus according to the workflow listed in Supplementary Table S4.

Homologs of the putative interaction partners were identified through the UniProtKB BLAST (available at: https://www.uniprot.org/blast/; accessed on 6th December 2021) with the “UniProtKB reference proteomes plus Swiss-Prot” database setting. Searches for homologs in *S.* *cerevisiae* were performed with the BLAST of the *Saccharomyces* *G*enome *D*atabase (SGD) (available at: https://www.yeastgenome.org/blast-sgd; accessed on 6th December 2021).

## Supplementary Information

Supplementary Information 1.

Supplementary Information 2.

## Data Availability

The mass spectrometry proteomics data have been deposited to the ProteomeXchange Consortium via the PRIDE partner repository^41^ with the dataset identifier PXD034217.
