# Supplementary material for: Establishment of in vivo proximity labeling with biotin using TurboID in the filamentous fungus Sordaria macrospora
Source: Sci Rep. 2022 Oct 22;12:17727. doi: 10.1038/s41598-022-22545-x (PMC9588061; doi:10.1038/s41598-022-22545-x)
Supplement: Supplementary file 1 — Supplementary Information 1. [file 41598_2022_22545_MOESM1_ESM.pdf]

Establishment of *in vivo* proximity labeling with biotin using TurboID in the filamentous fungus *Sordaria macrospora*

Lucas S. Hollstein[1], Kerstin Schmitt[2], Oliver Valerius[2], Gertrud Stahlhut[1] and Stefanie Pöggeler[1*]

[1] Georg-August-University of Göttingen, Institute of Microbiology and Genetics, Department of Genetics of Eukaryotic Microorganisms, Grisebachstr. 8, 37077 Göttingen, Germany; L.hollstein@stud.uni-goettingen.de (L.H.); gstahlh@gwdg.de (G.S.); spoegge@gwdg.de (S.P.)
[2] Georg-August-University of Göttingen, Institute of Microbiology and Genetics, Department of Molecular Microbiology and Genetics, Grisebachstr. 8, 37077 Göttingen, Germany; kschmit1@gwdg.de (K.S.); ovaleri@gwdg.de (O.V.)
* Correspondence: spoegge@gwdg.de; Tel.: +49-551-3924051

Note 1

Methods

Strains, media and growth conditions

Escherichia coli strain MACH1 (Thermo Fisher Scientific, C862003, Waltham, USA) was used for cloning and propagation of recombinant plasmids using standard culture conditions [1]. To generate recombinant plasmids by homologous recombination, we used *Saccharomyces cerevisiae* strain PJ69- 4A [2,3]. *S. macrospora* wild-type (wt) and Δsci1 mutant were transformed with recombinant plasmids as described previously [4]. All *S. macrospora* strains used in this study are listed in Table S1. The selection of recombinant strains was performed by supplementation of either hygromycin B (110 U/ml) or nourseothricin (50 µg/ml). *S. macrospora* strains were grown at 27 °C on liquid or solid biomalt maize medium (BMM), or *Sordaria* Westergaard's (SWG) fructification medium under continuous light conditions [5-7].

Generation of plasmids

All primers and template plasmids used for PCR amplifications are listed in Tables S2 and S3, respectively. Primers were synthesized by Sigma-Aldrich Chemie GmbH (Taufkirchen, Germany). Primers generated a 29-bp overhang at the PCR product which was together with *Xho*I linearized vector pRS-hyg [8] used for homologous recombination cloning in *S. cerevisiae* [2]. For the PCR amplification of the *N. crassa ccg1* promoter and *S. macrospora* promoter *Smxyl*, plasmid pHAN1 [9] and pPxyl-mng [10], respectively, was used as template with primer pairs pRSccg1/Pccg1-r and Pxylneon_fw/xyl1-r, respectively. To amplify the gene coding for *S. macrospora* codon optimized TurboID, plasmid pSmtBioID_pUC57 (ordered at GenScript) served as template for PCR. With primer pair SmtBioID-L_f/SmtBioID-r and SmtBioID-L-x_f/SmtBioID-r we amplified TurboID with the 11 aa linker for homologous recombination with *N. crassa ccg1* promoter and the *S. macrospora xyl* promoter, respectively. For PCR of TurboID ORF without the linker we used primer pairs SmtBioID_f/SmtBioID-r and SmtBioID-x_f/SmtBioID-r. The *Aspergillus nidulans trpC* terminator was amplified with primer pair

TrpC_F /TtrpC_pRS_r from plasmid p1783-1. Homologous recombination of promoter, TurboID ORF, and terminator resulted in plasmids pc-TurboID, pc-L-TurboID, px-TurboID, and px-L-TurboID, respectively (Figure 1 and Table S1). For fusing *Smsci1* N-terminally with the linker-TurboID under control of the *ccg1* promoter, we amplified *sci1* including the *ccg1* promoter with primer pair pRSccg1/sci1_tBioID-L_r from plasmid psci1GFP_nat [11]. The linker-TurboID fragment was amplified from plasmid pc-L-TurboID with the primer pair SmtBioID-L-f-2/TtrpC_pRS_r. Homologous recombination of the two PCR fragments with the *Xho*I linearized plasmid pRS-nat resulted in plasmid pc-sci1-L-TurboID. To generate plasmid pc-sci1-TurboID encoding the fusion protein without the linker, we used accordingly primer pair pRSccg1/sci1_tBioID_r2 with plasmid psci1GFP_nat as a template and primer pair SmtBioIDf-2/TtrpC_pRS_r with plasmid pc-TurboID as a template for PCR amplification. Homologous recombination into *Xho*I linearized plasmid pRS-nat resulted in plasmid pc-sci1-TurboID. For cloning the *Smsci1-linker-TurboID* fusion gene under control of the endogenous *sci1* promoter we amplified the promoter-*sci1* fragment from plasmid p5'sci1GFP_hyg [11] with primer pair rud3_5'f /sci1_tBioID-L_r and the *linker-TurboID* fragment from plasmid pc-L-TurboID with primer pair SmtBioID-L-f-2/TtrpC_pRS_r . Both fragments were integrated into the *Xho*I linearized plasmid pRS-nat and resulted in plasmid p5'-sci1-L-TurboID. For the fusion gene without the linker we used accordingly primer pair rud3_5'f/sci1_tBioID_r2 with plasmid p5'sci1GFP_hyg as a template and primer pair SmtBioIDf-2/TtrpC_pRS_r with plasmid pc-TurboID as a template for PCR. Homologous recombination with the *Xho*I linearized plasmid pRS-nat and resulted in plasmid p5'-sci1-TurboID (Figure 1 and Table S1).

Generation of single-spore isolates

Transformation of *S. macrospora* protoplasts produces heterokaryotic primary transformants (PTs). For the generation of homokaryotic strains, these PTs were crossed with the color spore mutant fus1-1, which exclusively produces brown ascospores due to a defective melanin biosynthesis [12]. Hybrid asci containing black and brown spores were isolated from the crossing front and plated on BMM medium supplemented with the appropriate antimycotic and 0.5 % sodium acetate, which induces germination of the spores.

Protein extraction from *S. macrospora*

For protein extraction, *S. macrospora* was grown in liquid BMM or SWG medium at 27 °C for 3 or 4 days, respectively. The mycelium was harvested, pressed dry between filter paper, shock frozen in liquid N_2, and stored at -80 °C until further use. Frozen mycelium was ground in a mortar under constant N_2 cooling. The mycelium powder was weighted, and lysis buffer (500 µL/g mycelia), as well as ~200 µL glass beads (Ø 0.25 – 0.5 mm) were added. Lysis was performed in the TissueLyser (Qiagen) bead mill at 30 Hz for 2:30 min. Afterwards, the samples were centrifuged for 10 min at 15 000 g and 4 °C to separate debris and solid particles from the crude protein extract.

Supplementary figures

A

ATGGGCGGCGGCGGCAGCGGCGGCGGCGGCTCCGGCGGCGGCGGCTCCGGCGGCGGCGGCAGCGGCGGCGGCGGCTCCGGC
GGCGGCGGCAGCGGCGGCGGCGGCTCCGACAAGGACAACACCGTCCCCCTCAAGCTCATCGCCCTCCTCGCCAACGGCGAGT
TCCACTCCGGCGAGCAGCTCGGCGAAACCCTCGGCATGTCCCGCGCCGCCATCAACAAGCACATCCAGACCCTCCGCGACTG
GGGCGTCGATGTCTTCACCGTCCCCGGCAAGGGCTACTCCCTCCCCGAGCCCATCCCCCTCCTCAACGCCAAGCAGATCCTCG
GCCAGCTCGACGGCGGCTCCGTCGCCGTCCTCCCCGTCGTCGATTCCACCAACCAGTACCTCCTCGACCGCATCGGCGAGCTA
AAGTCCGGCGACGCCTGCATCGCCGAGTACCAGCAGGCCGGCCGCGGCTCCCGCGGGCGCAAGTGGTTCTCCCCCTTCGGCG
CCAACCTCTACCTCTCCATCTTCTGGCGCCTCAAGCGCGGCCCCGCCGCCATCGGCCTCGGCCCCGTCATCGGCATCGTCATG
GCCGAGGCCCTCCGCAAGCTCGGCGCCGACAAGGTCCGCGTCAAGTGGCCCAACGACCTCTACCTCCAGGACCGCAAGCTCG
CCGGCATCCTCGTCGAACTCGCCGGCATCACCGGCGACGCCGCCCAGATCGTCATCGGCGCCGGCATCAACGTCGCCATGCG
CCGCGTCGAGGAGTCCGTCGTCAACCAGGGCTGGATCACCCTCCAGGAAGCCGGCATCAACCTCGACCGCAACACCCTCGCC
GCCACCCTCATCCGCGAGCTACGCGCCGCCCTCGAACTATTCGAGCAGGAAGGCCTCGCCCCCTACCTCCCCCGCTGGGAGAA
GCTCGACAACTTCATCAACCGCCCCGTCAAGCTCATCATCGGCGACAAGGAGATATTCGGCATCTCCCGCGGCATCGACAAG
CAGGGCGCCCTCCTCCTCGAACAGGACGGCGTCATCAAGCCCTGGATGGGCGGCGAGATCAGCCTCCGCTCCGCCGAGAAGA
AGCTCGCCTACCCCTACGACGTCCCCGACTACGCTTACCCATACGATGTGCCCGACTACGCCTACCCATACGATGTGCCAGAT
TACGCC TAA

B

MGGGGSGGGGSDKDNTVPLKLIALLANGEFHSGEQLGETLGMSRAAINKHIQTLRDWGVDVFTVPGKGYSLPEPIPLLNAKQILGQ
LDGGSVAVLPVVDSTNQYLLDRIGELKSGDACIAEYQQAGRGSRGRKWFSPFGANLYLSMFWRLKRGPAAIGLGPVIGIVMAEAL
RKLGADKVRVKWPNDLYLQDRKLAGILVELAGITGDAAQIVIGAGINVAMRRVEESVVNQGWITLQEAGINLDRNTLAATLIRELR
AALELFEQEGLAPYLPRWEKLDNFINRPVKLIIGDKEIFGISRGIDKQGALLLEQDGVIKPWMGGEISLRSAEKKLAYPYDVPDYA
YPYDVPDYA YPYDVPDYA-

Figure S1: Sequence of the *S. macrospora* codon optimized TurboID.

A) Nucleotide sequence and **B**) protein sequence. The 11 amino acid linker encoding the amino acid sequence MGGGGSGGGGS attached to N-terminus of TurboID is marked in green, the sequence of the 3 x HA Tag is marked in grey, and the sequence of TurboID in blue. Amino acids changed in TurboID relative to wild-type BirA are indicated in red.

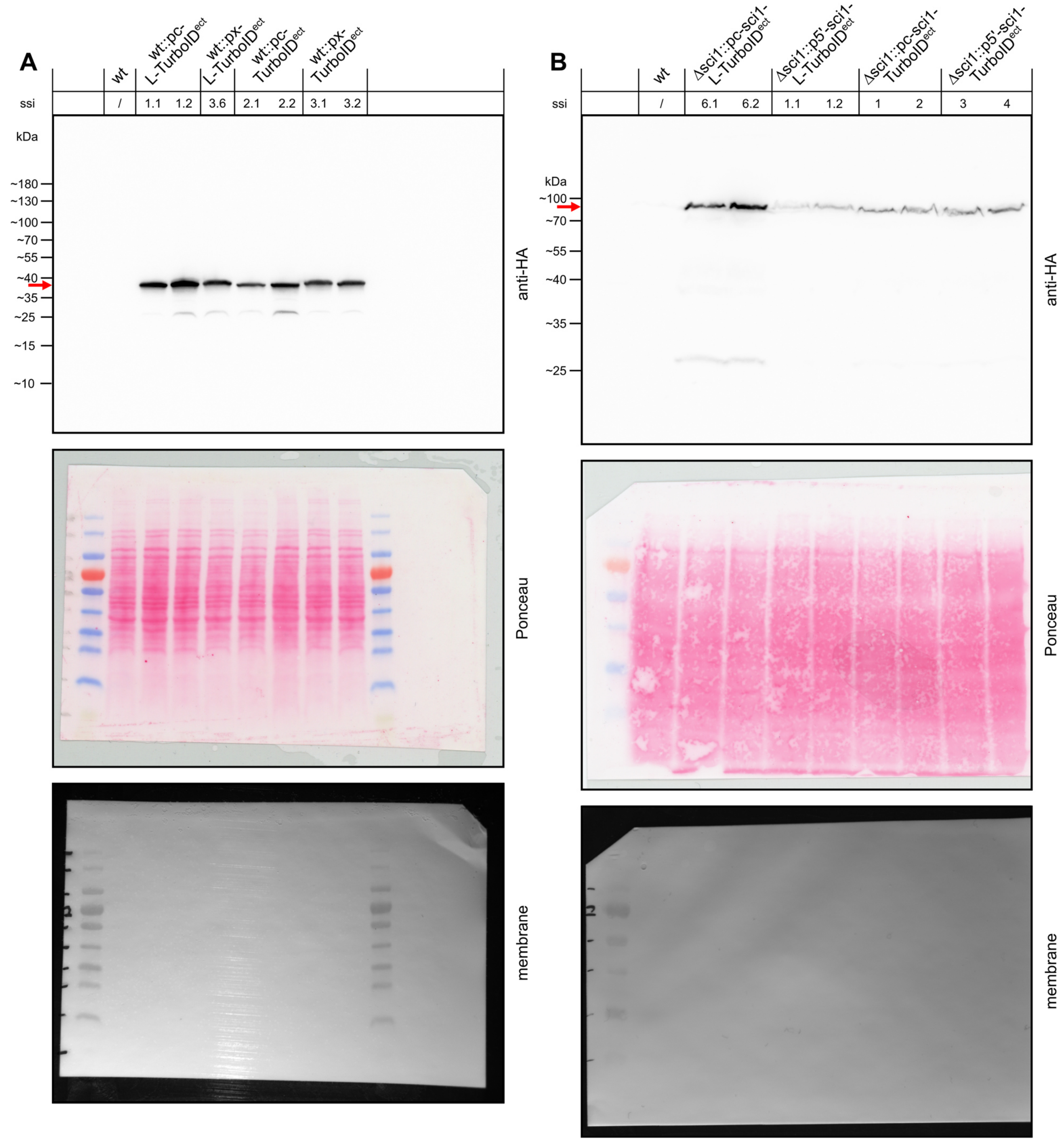

Figure S2: Western blot detection of TurboID constructs using an anti-HA antibody.

Expression of the **A**) free TurboID and **B**) SCI1-TurboID fusion proteins in *S. macrospora* wt and Δsci1 was determined by Western blot hybridization using a monoclonal anti-HA antibody, which detects the C-terminal 3xHA tag of TurboID. Red arrows indicate **A**) free TurboID (38.5 kDa) and **B**) SCI-TurboID fusion proteins (71.6 kDa). Expression of the constructs is controlled by either the *ccg1* overexpression promoter from *N. crassa* (pc), the native *sci1* promoter (p5'), or the xylose inducible *Smxyl* promoter (px) [13]. Untransformed wt was used as negative control. 18 µL of crude protein extract were loaded, and Ponceau red staining was used as loading control. Blots and Ponceau red staining in A and B show the identical membrane, exposure was identical for all parts of the gel. Pictures show whole blots without any recomposition. *S. macrospora* strains were grown in liquid BMM for 3 days at 27 °C. ect, ectopically integrated; ssi, single-spore isolate.

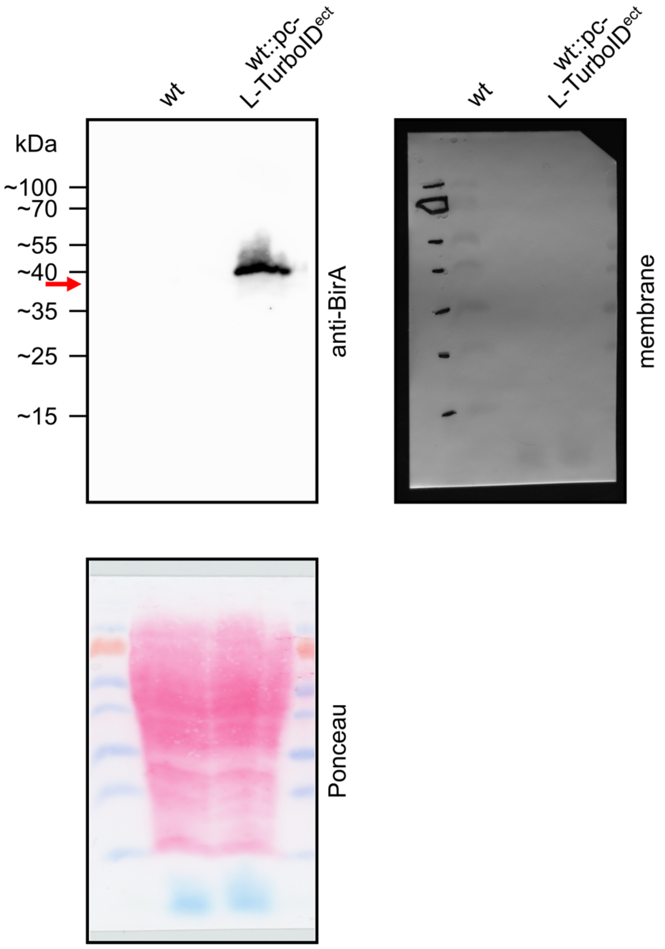

Figure S3: Western blot detection of free TurboID by an anti-BirA antibody.
Expression of free TurboID in *S. macrospora* was determined by Western blotting using a monoclonal anti-BirA antibody. The red arrow indicates free TurboID. Expression of free TurboID was controlled by the *ccg1* overexpression promoter from *N. crassa*. Untransformed wt was used as negative control. 20 µL of crude protein extract were loaded, and Ponceau red staining was used as loading control. For detection of the anti-BirA chemiluminescence, the membrane was orientated as shown in the picture of the membrane on the right. All pictures show the identical membrane and exposure was identical for all parts of the gel. Pictures show whole blots without any recomposition. The strains were grown in BMM medium for 3 days at 27 °C. ect, ectopically integrated

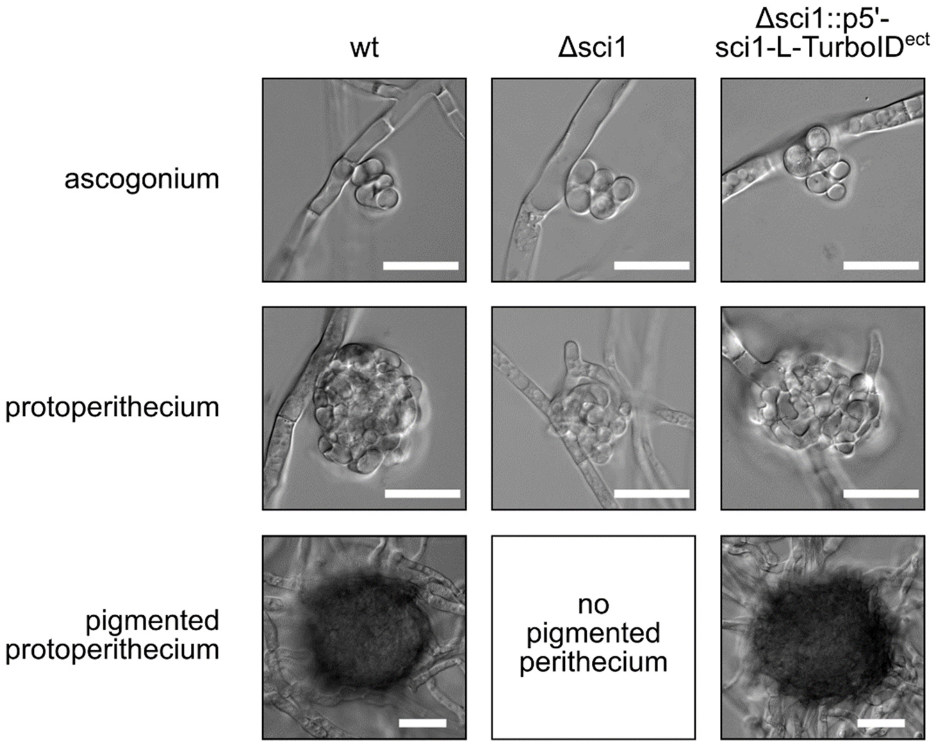

Figure S4: Sexual structures of wt, Δsci1 and Δsci1::p5’-sci1-L-TurboID^{ect}

Microscopic documentation of sexual structures of wt, Δsci1 and Δsci1::p5’-sci1-L-TurboID^{ect}. Development of sexual structures in Δsci1 is impaired and no pigmented perithecia are formed. This phenotype is complemented by the transformation of Δsci1 with *p5’-sci1-L-TurboID*. Scale bars are 20 µM.

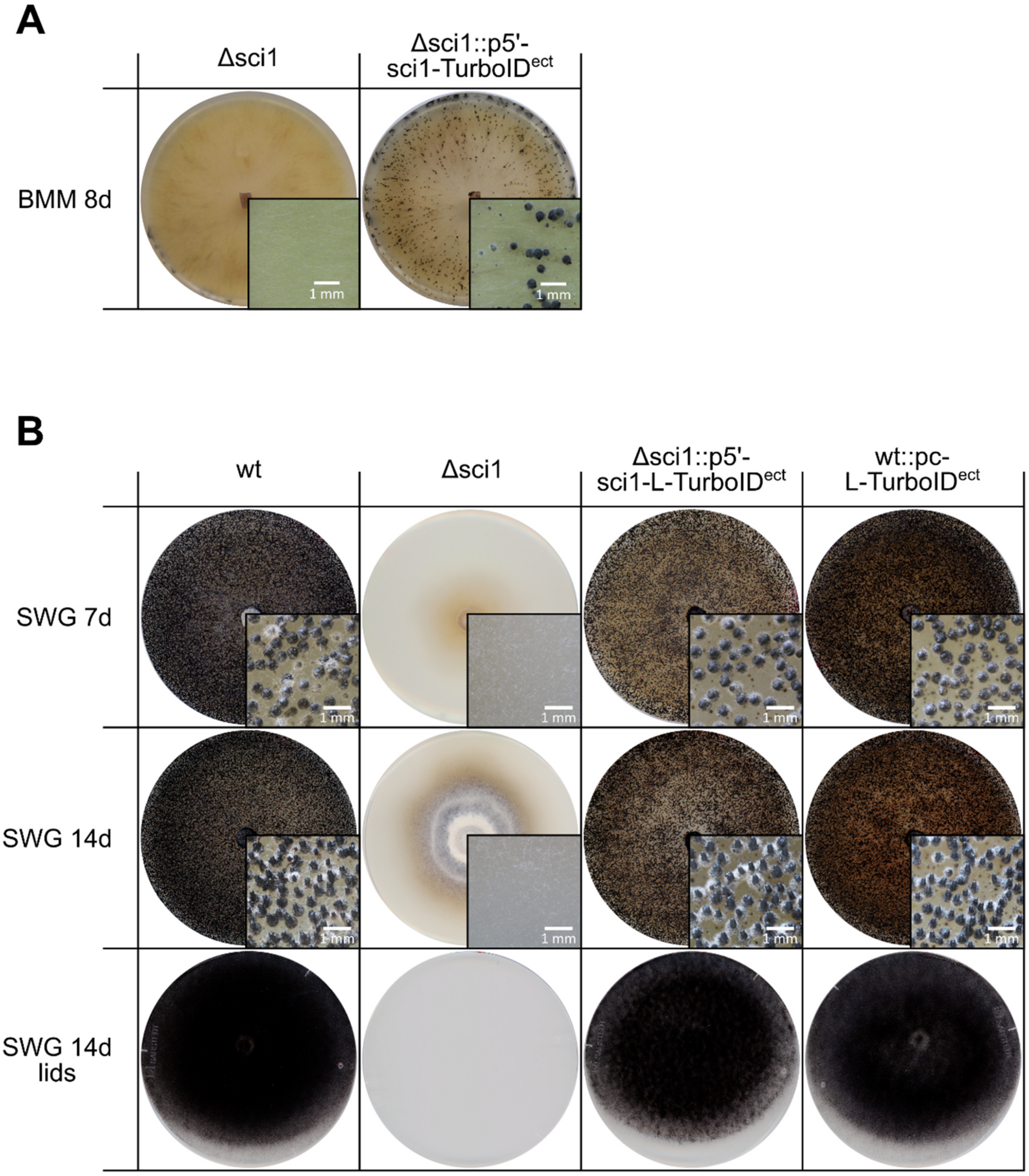

Figure S5: Complementation of Δsci1 and growth phenotypes of strains used in BioID.

A) The phenotype of the *S. macrospora* Δsci1 strain was complemented by transformation with the SCI1-TurboID fusion protein (without linker) controlled by the native *sci1* promoter (p5'-sci1-TurboID). The strains were grown for 8 days at 27 °C on BMM plates. Pictures of cultivated plates and magnifications were taken after 8 days. **B**) The phenotype of the *S. macrospora* Δsci1 strain was complemented by transformation with the SCI1-L-TurboID fusion protein (with linker) controlled by the native *sci1* promoter (p5'-sci1-L-TurboID). Overexpression of free L-TurboID controlled by the *ccg1* promoter resulted in wt-like growth and perithecia development. The strains were grown on SWG medium at 27 °C for 14 days. The lids with the discharged ascospores were documented after 14 d. Scale bars are indicated. ect, ectopically integrated

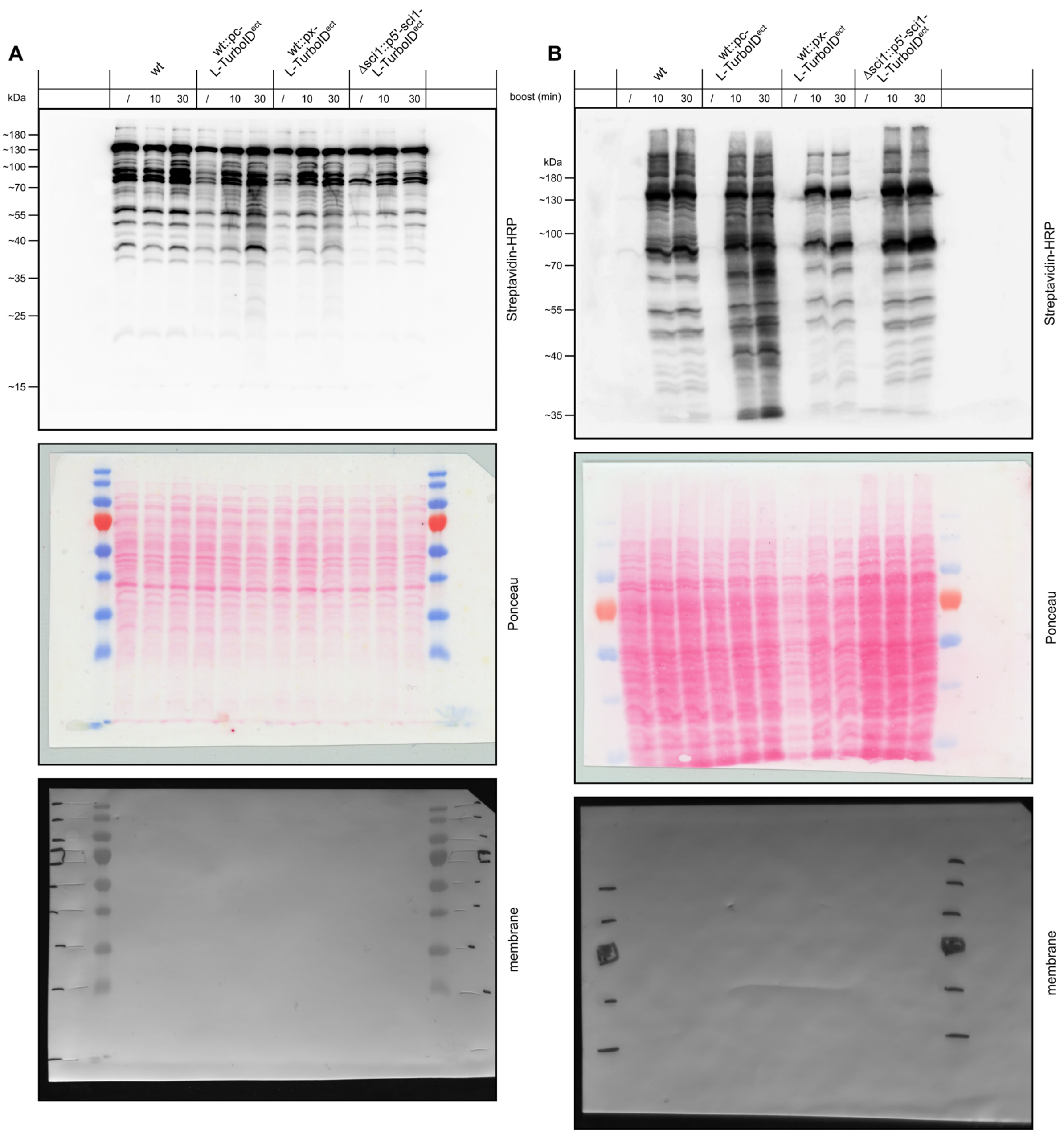

Figure S6: Western blot analysis of TurboID activity in recombinant *S. macrospora* strains.

The activity of free TurboID and SCI1-TurboID fusion proteins in *S. macrospora* was determined by Western blot-like hybridization using Streptavidin-HRP. Streptavidin binds biotinylated proteins, and HRP is used for signal detection via chemiluminescence. Expression of the constructs is controlled by either the *ccg1* overexpression promoter from *N. crassa* (pc), the native *sci1* promoter (p5'), or the xylose inducible *Smxyl* promoter (px) [13]. Untransformed wt shows endogenous biotinylation of *S. macrospora*. 2.25 µL crude protein extract were loaded onto the gel. Ponceau red staining was used as loading control. **A**) Strains were grown in liquid BMM medium for 3 days at 27 °C. Before harvest, BMM supplemented with biotin was added (final concentration = 410 nM) for 10 or 30 min. **B**) Strains were grown in liquid SWG medium (1 g/L arginine, without biotin) for 5 days at 27 °C. Shortly before harvest, SWG supplemented with biotin was added (final concentration = 410 nM) for 10 or 30 min. Blots and Ponceau red staining in A and B show the identical membrane, exposure was identical for all parts of the gel. Pictures show whole blots without any recomposition. ect, ectopically integrated

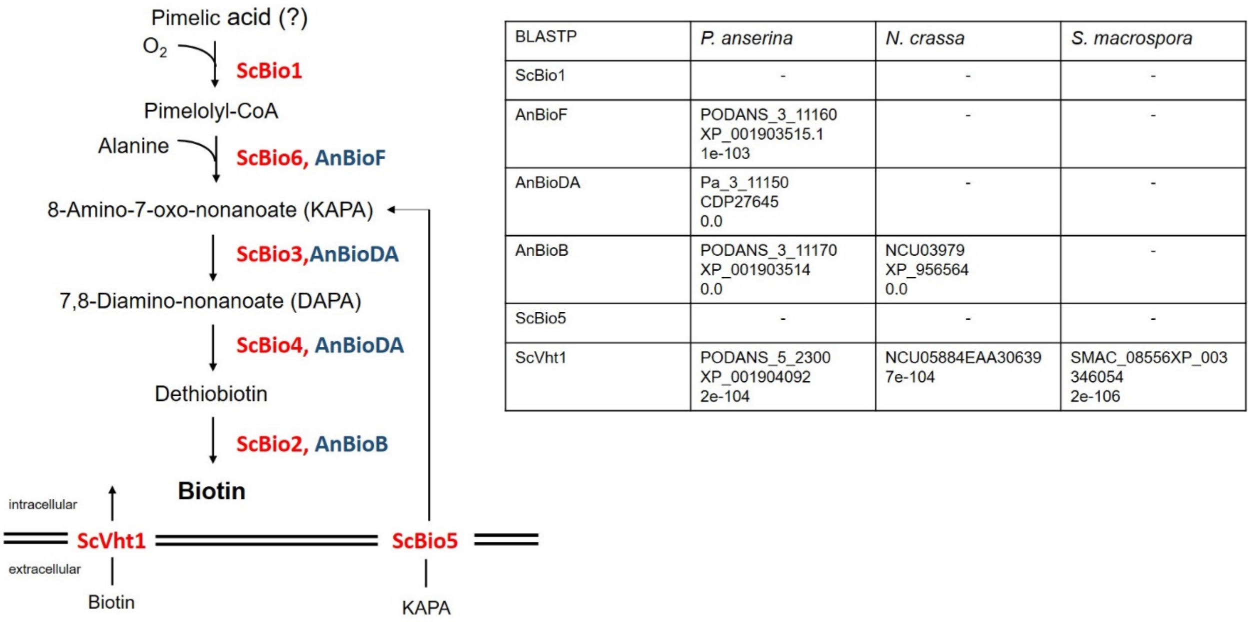

BLASTP	*P. anserina*	*N. crassa*	*S. macrospora*
ScBio1	-	-	-
AnBioF	PODANS_3_11160 XP_001903515.1 1e-103	-	-
AnBioDA	Pa_3_11150 CDP27645 0.0	-	-
AnBioB	PODANS_3_11170 XP_001903514 0.0	NCU03979 XP_956564 0.0	-
ScBio5	-	-	-
ScVht1	PODANS_5_2300 XP_001904092 2e-104	NCU05884EAA30639 7e-104	SMAC_08556XP_003 346054 2e-106

Figure S7: Biotin biosynthesis pathway in fungi.

In *Saccharomyces cerevisiae* the five steps of *de novo* biotin biosynthesis are carried out by ScBio1 (putative pimeloyl-CoA synthetase), ScBio6 (7-keto-8-aminopelargonic acid (KAPA) synthetase), ScBio3 (7,8-diamino-pelargonic acid aminotransferase (DAPA)), ScBio4 (dethiobiotin synthetase), and ScBio2 (biotin synthase). ScBio5 is a putative transmembrane protein involved in the biotin biosynthesis; responsible for uptake of 7-keto 8-aminopelargonic acid (KAPA) and ScVht1 a high-affinity plasma membrane H^+-biotin (vitamin H) symporter. In *S. cerevisiae* the origin of pimelic acid remains elusive (indicated by ?). In *Aspergillus nidulans*, the biotin synthesis pathway from the precursor pimeloyl-CoA onwards relies on three enzymes (AnBioF, AnBioDA, AnBioB) [14,15]. BALSTP searches with *S. cerevisiae* and *A. nidulans* biotin biosynthesis enzymes and transporters revealed that only a high-affinity plasma membrane H^+-biotin (vitamin H) symporter is encoded by *S. macrospora*. In contrast, the closely related species *Podospora anserina* encodes all enzymes required for *de novo* biotin biosynthesis while *Neurospora crassa* encodes only a biotin synthase.

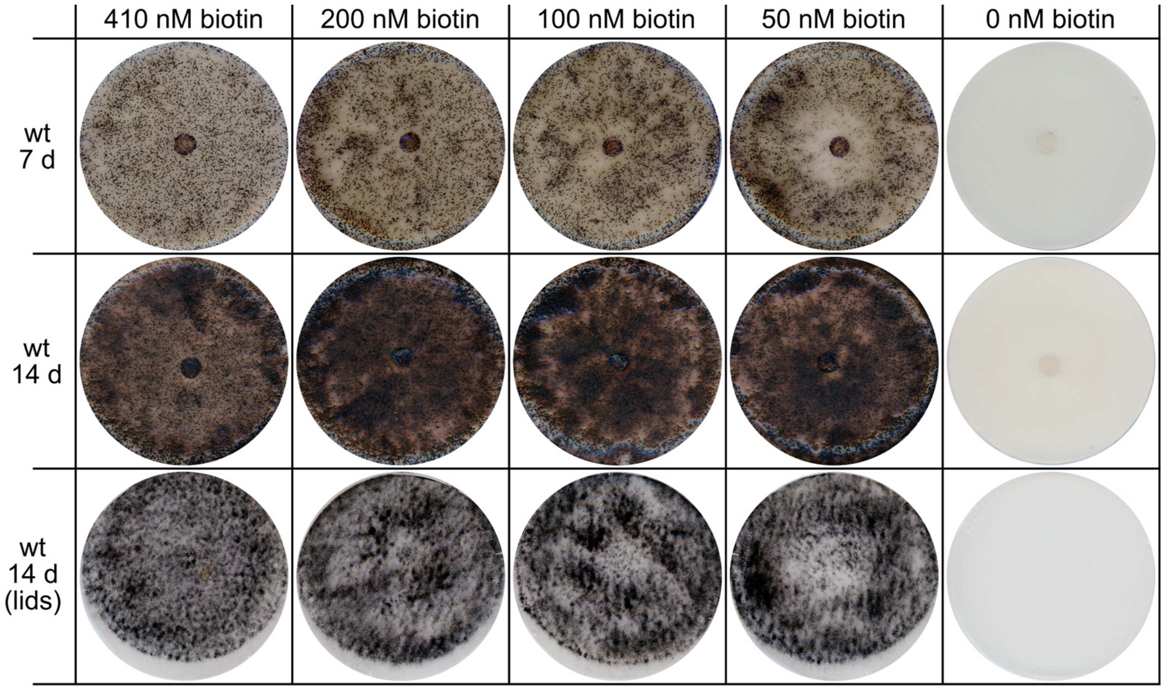

Figure S8: Growth of *S. macrospora* wt on SWG with different biotin concentrations.
S. macrospora wt was grown on SWG plates (40 mg/L arginine and 410, 200, 100, 50, or 0 nM biotin) for 14 days at 27 °C. Three replicates were inoculated with agar pieces from SWG without biotin. Pictures of cultivated plates were taken after 7 and 14 d. The lids containing discharged ascospores and demonstrating completion of the sexual cycle were documented after 14 d.

Supplementary tables

Table S1: *S. macrospora* strains used in this study.

S. macrospora strain	Genotype	Reference
wt (DSM997)	wild-type strain, black ascospores, fertile	DSMZ
fus1-1	mutated *fus1-1* gene, brown ascospores, fertile	[12]
Δsci1	Δsci1::*hyg*^{R}, black ascospores, sterile, ssi	[11]
wt::pc-TurboID^{ect}	ectopic integration of *pc-TurboID* into wt, *hyg*^{R}, fertile, ssi	this study
wt::pc-L-TurboID^{ect} ssi 1.7, 1.8, 1.9, 1.11	ectopic integration of *pc-L-TurboID* into wt, *hyg*^{R}, fertile, ssi	this study
wt::px-TurboID^{ect}	ectopic integration of *px-TurboID* into wt, *hyg*^{R}, fertile, ssi	this study
wt::px-L-TurboID^{ect}	ectopic integration of *px-L-TurboID* into wt, *hyg*^{R}, fertile, ssi	this study
Δsci1::p5'-sci1-TurboID^{ect}	ectopic integration of *p5'-sci1-TurboID* into Δsci1, *hyg*^{R}, *nat*^{R}, fertile, ssi	this study
Δsci1::p5'-sci1-L-TurboID^{ect} ssi 1.3, 1.6, 1.9, 1.10	ectopic integration of *p5'-sci1-L-TurboID* into Δsci1, *hyg*^{R}, *nat*^{R}, fertile, ssi	this study
Δsci1::px-sci1-TurboID^{ect}	ectopic integration of *px-sci1-TurboID* into Δsci1, *hyg*^{R}, *nat*^{R}, fertile, ssi	this study
Δsci1::px-sci1-L-TurboID^{ect}	ectopic integration of *px-sci1-L-TurboID* into Δsci1, *hyg*^{R}, *nat*^{R}, fertile, ssi	this study

ect, ectopically integrated; *p5'*, native *sci1* promoter; *pc*, promoter of the *clock-controlled gene 1* (*ccg1*) from *N. crassa* for overexpression; *px*, xylose inducible promoter (*Smxyl*) of the *beta-xylanase* gene (*SMAC_08023*) from *S. macrospora* [13]; L, MGGGGSGGGGS linker attached to N-terminus of TurboID; *nat*^{R}, nourseothricin resistant; *hyg*^{R}, hygromycin resistant; ssi, single-spore isolates.

Table S2: Primers used in this study.

Oligonucleotide	Sequence
pRSccg1	*GTAACGCCAGGGTTTTCCCAGTCACGACG* TAGAAGGAGCAGTCCATCTG
Pccg1-r	TTTGGTTGATGTGAGGGGTT
Pxylneon_fw	*GTAACGCCAGGGTTTTCCCAGTCACGACG* GAACCTTCTCTTCTCCATTT
xyl1-r	GTTGGCGGTTTCTGGTTAGGCC
TtrpC_pRS_r	*GCGGATAACAATTTCACACAGGAAACAGC* TCGAGTGGAGATGTGGAGTGG
TrpC_F	GATCCACTTAACGTTACTGAAATCATCAAA
SmtBioID-L_f	*CACTTTCACAACCCCTCACATCAACCAAA* GATATCATGGGCGGCGGCGGCAG
SmtBioID-r	*TTGATGATTTCAGTAACGTTAAGTGGATC* TTAGGCGTAATCTGGCACATC
SmtBioID-L-x_f	*CCCTCTAGGCCTAACCAGAAACCGCCAAC* GATATCATGGGCGGCGGCGGCAG
SmtBioID_f	*CACTTTCACAACCCCTCACATCAACCAAA* ATGAAGGACAACACCGTCCCCCTC
SmtBioID-x_f	*CCCTCTAGGCCTAACCAGAAACCGCCAAC* ATGAAGGACAACACCGTCCCCCTC
sci1_tBioID-L_r	*CCGCCGCCGCCGCTGCCGCCGCCGCCCAT* GGAAACCGGCGGCTGTGGTAC
SmtBioID-L-f-2	ATGGGCGGCGGCGGCAGCGGCGGC
sci1_tBioID_r2	*AGCTTGAGGGGGACGGTGTTGTCCTTCAT* GGAAACCGGCGGCTGTGGTAC
SmtBioIDf-2	ATGAAGGACAACACCGTCCCC
rud3_5’f	*GTAACGCCAGGGTTTTCCCAGTCACGACG* GCTAGCTATCAACATCATCG

Overhangs for homologous recombination in yeast are in italics and underlined.

Table S3: Plasmids used in this study.

Plasmid	Characteristics	Reference
pSmtBioID_pUC57	*L-TurboID::3xHA S. macrospora codon optimized*	GenScript
pRS-hyg	amp^R, *ura3*, hyg^R	[8]
pRS-nat	amp^R, *ura3*, nat^R	[16]
pPxyl-mng	amp^R, *ura3*, nat^R *px::mng::TtrpC*	[10]
p1783-1	amp^R, *ura3*, hyg^R, *Pgpd::egfp::TtrpC*	[17]
pHAN1	amp^R, *his-3*, *pc::ha*	[9]
psci1GFP_nat	amp^R, *ura3, natR, pc::sci1::egfp::TtrpC*	[11]
p5'sci1GFP_hyg	amp^R, *ura3*, nat^R, *5'sci1::sci1::egfp::TtrpC*	[11]
p5'-sci1-TurboID	*p5'::sci1-TurboID::3xHA::TtrpC*, nat^R	this study
p5'-sci1-L-TurboID	*p5'::sci1-L-TurboID::3xHA::TtrpC*, nat^R	this study
pc-sci1-TurboID	*pc::sci1-TurboID::3xHA::TtrpC*, hyg^R	this study
pc-sci1-L-TurboID	*pc::sci1-L-TurboID::3xHA::TtrpC*, hyg^R	this study
pc-TurboID	*pc::TurboID::3xHA::TtrpC*, hyg^R	this study
pc-L-TurboID	*pc::L-TurboID::3xHA::TtrpC*, hyg^R	this study
px-TurboID	*px::TurboID::3xHA::TtrpC*, hyg^R	this study
px-L-TurboID	*px::L-TurboID::3xHA::TtrpC*, hyg^R	this study

The TurboID sequence was codon optimized according to the codon usage table of *S. macrospora* [18]. *p5'*, native *sci1* promoter; *pc*, promoter of the *clock-controlled gene 1* (*ccg1*) from *N. crassa* for overexpression; *px*, xylose inducible promoter (*Smxyl*) of the *beta-xylanase* gene (*SMAC_08023*) from *S. macrospora* [13]; L, MGGGGSGGGGS linker attached to N-terminus of TurboID; *3xHA*, triple HA-tag at the C-terminus of TurboID; *TtrpC*, terminator of the anthranilate synthase gene from *A. nidulans*; nat^R, nourseothricin resistance; hyg^R, hygromycin resistance

Table S4: Workflow for analysis of BioID experiments in Perseus.

#	Command	Description
1	Generic matrix upload	proteinGroups.txt<br>Main: LFQ intensities<br>Numerical: MS/MS count
2	Filter rows based on categorical column	Remove rows containing "+" for columns "only identified by site"; "Reverse" and "potential contaminant"
3	Transform	LFQ intensities: log2(x)
4	Categorical annotation	Create group with "control" and "Δsci1" subgroups
5	Analysis	Multi scatter plot
6	Filter rows based on valid values	50 % valid values in total
7	Replace missing values from normal distribution	Mode: Total matrix
8	Tests	Two-sample test<br>Student's T-test, s0=2; FDR = 0.01
9	Repeat step 7-8 four times<br>Manually select significant hits with $\log_2$(difference) ≥ 2<br>in all four imputations and export to new matrix	
10	Replace imputed values by NaN	
11	Copy and paste matrix into Excel for further evaluation	

Table S5: BLAST results of significantly enriched proteins in BioID experiments.
Homologs of uncharacterized proteins from BioID experiments were identified using the UniProtKB BLAST with the "UniProtKB reference proteomes and Swiss-Prot" database. Identifications in *Neurospora crassa, Madurella mycetomatis, Neursospora tetrasperma* and *Sordaria macrospora.*

BLAST results					
**SMAC ID**	**gene**	**organism**	**accession number**	**e-value**	**identity**
SMAC_00725	biotin apo-protein ligase	*N. crassa*	Q7S6S3	0.0	77.6 %
SMAC_00877	SmMOB3	*S. macrospora*	[19]		
SMAC_01219	NAD-dependent protein deacetylase	*S. macrospora*	F7VQ70	0.0	94.7 %
SMAC_02580	PRO22	*S. macrospora*	[8]		
SMAC_03234	pre-mRNA-splicing factor SPF27	*M. mycetomatis*	A0A175W8J4	6.3e-96	65.3 %
SMAC_03446	PP2A phosphatase activator (PTPA1)	*S. macrospora*	[20]		
SMAC_04678	SmPP2Ac1	*S. macrospora*	Beier et al. (2016)[20]		
SMAC_05070	signal recognition particle 54 kDa protein	*S. macrospora*	F7W2K7	0.0	100 %
SMAC_05559	SCI1	*S. macrospora*	[11]		
SMAC_08794	PRO11	*S. macrospora*	[21]		

References

1 Sambrook, J., Fritsch, E. & Maniatis, T. *Molecular cloning: a laboratory manual*. 2nd Ed. edn, (Cold Spring Harbor Laboratory, Cold Spring Habor, 2001).

2 Colot, H. V., Park, G., Turner, G. E., Ringelberg, C., Crew, C. M. *et al.* A high-throughput gene knockout procedure for Neurospora reveals functions for multiple transcription factors. *Proc. Natl. Acad. SCI. U.S.A.* **103**, 10352-10357, doi:10.1073/pnas.0601456103 (2006).

3 James, P., Halladay, J. & Craig, E. A. Genomic libraries and a host strain designed for highly efficient two-hybrid selection in yeast. *Genetics* **144**, 1425-1436 (1996).

4 Walz, M. & Kück, U. Transformation of *Sordaria macrospora* to hygromycin B resistance: characterization of transformants by electrophoretic karyotyping and tetrad analysis. *Curr. Genet.* **29**, 88-95, doi:10.1007/bf00313198 (1995).

5 Elleuche, S. & Pöggeler, S. Visualization of peroxisomes via SKL-tagged DsRed protein in *Sordaria macrospora*. *Fungal Geneti. Rep.* **55**, 8-12 (2008).

6 Esser, K. *Cryptogams: Cyanobacteria, Algae, Fungi, Lichens*. (CUP Archive, 1982).

7 Nowrousian, M., Ringelberg, C., Dunlap, J. C., Loros, J. J. & Kück, U. Cross-species microarray hybridization to identify developmentally regulated genes in the filamentous fungus *Sordaria macrospora*. *Mol. Genet. Genom.* **273**, 137-149, doi:10.1007/s00438-005-1118-9 (2005).

8 Bloemendal, S., Bernhards, Y., Bartho, K., Dettmann, A., Voigt, O. *et al.* A homologue of the human STRIPAK complex controls sexual development in fungi. *Mol. Microbiol.* **84**, 310–323, doi:10.1111/j.1365-2958.2012.08024.x (2012).

9 Kawabata, T. & Inoue, H. Detection of physical interactions by immunoprecipitation of FLAG- and HA-tagged proteins expressed at the his-3 locus in *Neurospora crassa*. *Fungal Genet. Newsl.* **54**, 5-8 (2007).

10 Werner, A., Otte, K. L., Stahlhut, G. & Pöggeler, S. Establishment of the monomeric yellow-green fluorescent protein mNeonGreen for life cell imaging in mycelial fungi. *AMB Express* **10**, 222, doi:10.1186/s13568-020-01160-x (2020).

11 Reschka, E. J., Nordzieke, S., Valerius, O., Braus, G. H. & Pöggeler, S. A novel STRIPAK complex component mediates hyphal fusion and fruiting-body development in filamentous fungi. *Mol. Microbiol.* **110**, 513–532, doi:10.1111/mmi.14106 (2018).

12 Nowrousian, M., Teichert, I., Masloff, S. & Kück, U. Whole-genome sequencing of *Sordaria macrospora* mutants identifies developmental genes. *G3-Genes Genome Genet.* **2**, 261–270, doi:10.1534/g3.111.001479 (2012).

13 Bloemendal, S., Löper, D., Terfehr, D., Kopke, K., Kluge, J. *et al.* Tools for advanced and targeted genetic manipulation of the β-lactam antibiotic producer *Acremonium chrysogenum*. *J. Biotechnol.* **169**, 51–62, doi:10.1016/j.jbiotec.2013.10.036 (2014).

14 Magliano, P., Flipphi, M., Sanglard, D. & Poirier, Y. Characterization of the *Aspergillus nidulans* biotin biosynthetic gene cluster and use of the bioDA gene as a new transformation marker. *Fungal Genet. Biol.* **48**, 208-215, doi:https://doi.org/10.1016/j.fgb.2010.08.004 (2011).

15 Perli, T., Wronska, A. K., Ortiz-Merino, R. A., Pronk, J. T. & Daran, J. M. Vitamin requirements and biosynthesis in *Saccharomyces cerevisiae*. *Yeast (Chichester, England)* **37**, 283-304, doi:10.1002/yea.3461 (2020).

16 Klix, V., Nowrousian, M., Ringelberg, C., Loros, J. J., Dunlap, J. C. *et al.* Functional characterization of MAT1-1-specific mating-type genes in the homothallic ascomycete *Sordaria macrospora* provides new insights into essential and nonessential sexual regulators. *Eukaryot. Cell* **9**, 894-905, doi:10.1128/ec.00019-10 (2010).

17 Pöggeler, S., Masloff, S., Hoff, B., Mayrhofer, S. & Kück, U. Versatile EGFP reporter plasmids for cellular localization of recombinant gene products in filamentous fungi. *Curr. Genet.* **43**, 54-61, doi:10.1007/s00294-003-0370-y (2003).

18 Nowrousian, M., Stajich, J. E., Chu, M., Engh, I., Espagne, E. *et al.* De novo assembly of a 40 Mb eukaryotic genome from short sequence reads: *Sordaria macrospora*, a model organism for fungal morphogenesis. *PLoS Genet.* **6**, e1000891, doi:10.1371/journal.pgen.1000891 (2010).

19 Bernhards, Y. & Pöggeler, S. The phocein homologue SmMOB3 is essential for vegetative cell fusion and sexual development in the filamentous ascomycete *Sordaria macrospora*. *Curr. Genet.* **57**, 133–149, doi:10.1007/s00294-010-0333-z (2011).

20 Beier, A., Teichert, I., Krisp, C., Wolters, D. A. & Kück, U. Catalytic subunit 1 of protein phosphatase 2A is a subunit of the STRIPAK complex and governs fungal sexual development. *mBio* **7**, e00870-00816, doi:10.1128/mBio.00870-16 (2016).

21 Pöggeler, S. & Kück, U. A WD40 repeat protein regulates fungal cell differentiation and can be replaced functionally by the mammalian homologue Striatin. *Eukaryot. Cell* **3**, 232–240, doi:10.1128/EC.3.1.232-240.2004 (2004).
